# Ozone source apportionment during peak summer events over southwestern Europe

**DOI:** 10.5194/acp-19-5467-2019

**Published:** 2019-04-25

**Authors:** María Teresa Pay, Gotzon Gangoiti, Marc Guevara, Sergey Napelenok, Xavier Querol, Oriol Jorba, Carlos Pérez García-Pando

**Affiliations:** 1 Earth Sciences Department, Barcelona Supercomputing Center, BSC, c/Jordi Girona, 29, 08034 Barcelona, Spain; 2 Department of Chemical and Environmental Engineering, University of the Basque Country UPV/EHU, ETSI-Bilbao School of Engineering, Alameda de Urquijo s/n, 48013 Bilbao, Spain; 3 United States Environmental Protection Agency, Research Triangle Park, NC, USA; 4 Institute of Environmental Assessment and Water Research, IDAEA-CSIC, c/Jordi Girona, 18–26, 08034 Barcelona, Spain

## Abstract

It is well established that in Europe, high O_3_ concentrations are most pronounced in southern/Mediterranean countries due to the more favourable climatological conditions for its formation. However, the contribution of the different sources of precursors to O_3_ formation within each country relative to the imported (regional and hemispheric) O_3_ is poorly quantified. This lack of quantitative knowledge prevents local authorities from effectively designing plans that reduce the exceedances of the O_3_ target value set by the European air quality directive. O_3_ source attribution is a challenge because the concentration at each location and time results not only from local biogenic and anthropogenic precursors, but also from the transport of O_3_ and precursors from neighbouring regions, O_3_ regional and hemispheric transport and stratospheric O_3_ injections. The main goal of this study is to provide a first quantitative estimation of the contribution of the main anthropogenic activity sectors to peak O_3_ events in Spain relative to the contribution of imported (regional and hemispheric) O_3_. We also assess the potential of our source apportionment method to improve O_3_ modelling. Our study applies and thoroughly evaluates a countrywide O_3_ source apportionment method implemented in the CALIOPE air quality forecast system for Spain at high resolution (4 × 4 km^2^) over a 10-day period characterized by typical summer conditions in the Iberian Peninsula (IP). The method tags both O_3_ and its gas precursor emissions from source sectors within one simulation, and each tagged species is subject to the typical physico-chemical processes (advection, vertical mixing, deposition, emission and chemistry) as the actual conditions remain unperturbed. We quantify the individual contributions of the largest NO_*x*_ local sources to high O_3_ concentrations compared with the contribution of imported O_3_. We show, for the first time, that imported O_3_ is the largest input to the ground-level O_3_ concentration in the IP, accounting for 46 %–68 % of the daily mean O_3_ concentration during exceedances of the European target value. The hourly imported O_3_ increases during typical northwestern advections (70 %–90 %, 60–80 μg m^−3^), and decreases during typical stagnant conditions (30 %–40 %, 30–60 μg m^−3^) due to the local NO titration. During stagnant conditions, the local anthropogenic precursors control the O_3_ peaks in areas downwind of the main urban and industrial regions (up to 40 % in hourly peaks). We also show that ground-level O_3_ concentrations are strongly affected by vertical mixing of O_3_-rich layers present in the free troposphere, which result from local/regional layering and accumulation, and continental/hemispheric transport. Indeed, vertical mixing largely explains the presence of imported O_3_ at ground level in the IP. Our results demonstrate the need for detailed quantification of the local and remote contributions to high O_3_ concentrations for local O_3_ management, and show O_3_ source apportionment to be an essential analysis prior to the design of O_3_ mitigation plans in any non-attainment area. Achieving the European O_3_ objectives in southern Europe requires not only ad hoc local actions but also decided national and European-wide strategies.

## 1 Introduction

Tropospheric ozone (O_3_) is an air pollutant of major public concern as it harms human health (WHO, 2013) and sensitive vegetation (Booker et al., 2009), and contributes to climate change (Jacob and Winner, 2009). O_3_ is formed in the atmosphere through non-linear photochemical reactions among carbon monoxide (CO), peroxy radicals generated by the photochemical oxidation of volatile organic compounds (VOCs) and nitrogen oxides (NO_*x*_) (Crutzen, 1974). Therefore, meteorological stagnation, high temperatures, high solar radiation and low precipitation enhance tropospheric O_3_ formation (Demuzere et al., 2009; Otero et al., 2016). Atmospheric circulation also controls the short- and long-range transport of O_3_ affecting its lifetime in the atmosphere (Monks et al., 2015). For example, the transport of precursors emitted in urban and industrialized areas may cause O_3_ production downwind (Holloway et al., 2003).

According to the European Environmental Agency (EEA) around 95 %–98 % of the population in Europe were exposed to O_3_ concentrations that exceeded the guidelines of the World Health Organization (WHO) during 2013–2015 period (EEA, 2017). These guidelines establish a maximum daily 8 h averaged (MDA8) O_3_ concentration of 100 μg m^−3^ never to be exceeded. The European air quality directive (2008/50/EC) is less restrictive as it sets an O_3_ target value of 120 μg m^−3^ for the MDA8 concentration, which can be exceeded up to 25 days per calendar year averaged over 3 years.

Southern European countries around the Mediterranean Basin are particularly exposed to exceedances of the O_3_ target value in summer due to the influence of frequent anticyclonic and clear-sky conditions that favour photochemical O_3_ formation in the troposphere (EEA, 2017). In addition, its geographic location also makes the basin a receptor of the long-range transport of pollution from Europe, Asia and even North America (Lelieveld et al., 2002; Gerasopoulos, 2005). The importance of long-range transport on surface O_3_ has been studied in the Mediterranean Basin, indicating that the emission sources within the basin have a dominating influence on surface O_3_, whereas remote sources are more important than local sources for O_3_ mixing ratios at higher altitudes (Richards et al., 2013; Safieddine et al., 2014). Recent studies have suggest that the upper O_3_-rich air masses could increase the surface O_3_ concentration in the Mediterranean Basin (Kalabokas et al., 2017; Querol et al., 2018). Further detailed and quantitative studies on the mechanism linking the upper O_3_-rich layer with increases in the ground-level O_3_ concentration in episodes require further clarification particularly regarding the contribution of O_3_ transported at regional and hemispheric scales.

Several studies in the Iberian Peninsula (IP) have addressed the causes of O_3_ episodes by looking at the circulation of air masses (Millán, 2014, and references therein). In the Atlantic region, the blocking anticyclones over western Europe favour the inter-regional transport of O_3_ in the area and its accumulation for several days during the most severe episodes (Alonso et al., 2000; Gangoiti et al., 2002, 2006; Valdenebro et al., 2011; Saavedra et al., 2012; Monteiro et al., 2016). Conversely, on the Mediterranean coast, the typical summer synoptic meteorological conditions with a lack of strong synoptic advection, combined with the orographic characteristics and the sea and land breezes, favour episodes where high levels of O_3_ are accumulated by recirculation of air masses loaded with O_3_ precursors (Millán et al., 1997, 2000; Toll and Baldasano, 2000; Gangoiti et al., 2001; Pérez et al., 2004; Jiménez et al., 2006; Gonçalves et al., 2009; Millán, 2014; Querol et al., 2017, 2018). The coupling between synoptic and mesoscale processes governing the levels of O_3_ in the western Mediterranean Basin need further research in order to understand the O_3_ intercontinental contribution. Furthermore, based on our understanding there is a lack of research quantifying the contribution of the activity sources to the O_3_ local formation during peak events in this region.

O_3_ analyses in the western Mediterranean Basin show that regional background O_3_ levels have remained high without significant changes (EEA, 2017; EMEP-CCC, 2016; Querol et al., 2016). However, they have increased at traffic and urban background sites (EEA, 2017; Querol et al., 2016; Sicard et al., 2016; Saiz-Lopez et al., 2017). The reasons behind the urban O_3_ upward trend are not clear yet due to the complex VOC-NO_*x*_ regime; part of the O_3_ increase may have resulted from the reduction of NO emissions relative to NO_2_ and therefore to a lower NO titration effect in VOC-limited regimes. The most intense O_3_ events in the last decade, measured by the number of exceedances of the O_3_ target value are recorded over areas downwind of large urban and industrial hot spots (Monterio et al., 2012; Querol et al., 2016; EEA, 2016). Overall, a number of these types of O_3_ events occur in June–July and during summer heat waves (i.e. 2003 and 2015).

According to the European air quality directive, in zones exceeding the O_3_ target value, EU member states must develop plans to attain compliance by reducing the emission of O_3_ precursors. Abatement of the tropospheric O_3_ concentration in the western Mediterranean Basin has been insufficient thus far (Querol et al., 2018). Effective planning requires an accurate quantitative knowledge of the sources of these precursors and their respective contributions to the exceedances of the O_3_ target value (Querol et al., 2016; Borrego et al., 2016). However, source attribution of surface O_3_ concentrations remains a challenge, because the concentration at each location and time results not only from local biogenic and anthropogenic precursors, but also from the transport of O_3_ and its precursors from neighbouring regions, O_3_ hemispheric transport (UNECE, 2010) and stratospheric O_3_ injections (Monks et al., 2015).

At present, there are no methods based on observations that distinguish the origin of O_3_. Despite their inherent uncertainties, chemical transport models (CTMs) allow for the apportionment of the contribution of any source (by sector and/or region) to O_3_ concentrations. The most widely used approach is the “brute force” method, which consists of running an ensemble of simulations zeroing out the sources one by one and then comparing them with a baseline simulation that accounts for all of the sources. Several O_3_ source apportionment studies at a European scale have applied the brute force method to quantify the contribution of one or two emission sectors. For example, road transport emissions using the EMEP model (Reis et al., 2000), biogenic and anthropogenic emissions using the Polyphemus model (Sartelet et al., 2012), transport-related emissions including road transport, shipping and aviation using the WRF-CMAQ model (TRANSPHORM, 2014), and ship emissions with CAMx (Aksoyoglu et al., 2016). Brute force is simple to implement, as it does not require additional coding in the CTM. However, as it quantifies the contribution of each source based on its absence, it does not reproduce actual atmospheric conditions; therefore, it is susceptible to inaccuracies in the prediction of O_3_ peaks under non-linear regimes (Cohan and Napelenok, 2011). Actually, brute force is not suitable for retrieving source contributions when the relationship between emissions and concentrations is non-linear, but it is useful for analysing the concentration responses to emission abatement scenarios (Clappier et al., 2017).

Recently, CTMs include algorithms that tag multiple pollutants by source (region and/or sector) all the way through the pollutant’s lifetime, from emission to deposition. This integrated source apportionment approach has several advantages. First, it allows for the identification of the main sources contributing to high O_3_ levels under actual atmospheric conditions, which is a preliminary step towards designing refined and efficient emission abatement scenarios. Second, as we show below, it supports enhanced model evaluation and therefore potential model improvements by identifying problems in emission estimates (sectors or regions) or chemical boundary conditions. The Integrated Source Apportionment Method (ISAM) within the Community Multiscale Air Quality (CMAQ) model has shown promising results for O_3_ tagging, exhibiting less noise in locations where brute force results are demonstrably inaccurate (Kwok et al., 2013, 2015). Recent ISAM experiments have quantified that the contribution of traffic in the cities of Madrid and Barcelona to the daily O_3_ peaks downwind of the urban areas is particularly significant (up to 80–100 μg m^−3^) (Valverde et al., 2016a). O_3_ tagging methods are also included in other regional and global models applied over Europe (Karamchandani et al., 2017; Butler et al., 2018).

The integrated source apportionment tools combined with high-resolution emission and meteorological models can help unravelling the sources responsible for peak summer events of O_3_ in the western Mediterranean Basin. Quantifying the contribution of emission sources during acute O_3_ episodes is a prerequisite for the design of future mitigation strategies in the region. In this framework, the main goal of this study is to provide a first quantitative estimation of the contribution of the main anthropogenic activity sectors compared to the imported concentration (regional and hemispheric) to peak O_3_ events in Spain. We also assess the potential of our source apportionment method to improve O_3_ modelling. Our study applies, for the first time, a countrywide O_3_ source apportionment at high resolution over the IP during the period between 21 and 31 July 2012, which is representative of the typical summer synoptic conditions in the region. We use the CMAQ-ISAM within the CALIOPE air quality forecast system for Spain (http://www.bsc.es/caliope/es, last access: 4 April 2019), which runs at a horizontal resolution of 4 × 4 km^2^ over the IP. The system is fed by the HERMESv2.0 emission model, which provides disaggregated emissions based on local information and state-of-the-art bottom-up approaches for the most prevalent pollution sectors.

The paper is organized as follows. In Sect. 2 we introduce the CALIOPE system, the set-up of ISAM and the HERMESv2.0 emission model for O_3_ source apportionment studies, and the methodology used to quantify the evaluation of the model. In Sect. 3 we demonstrate the representativeness of the selected episode, we evaluate the model and we provide an analysis of the source-sector contribution to Spanish O_3_ under the different synoptic patterns occurring during the study period. In Sect. 4, we discuss our findings, the regulatory implications and future research.

## 2 Methodology

### 2.1 Air quality model

We used the CALIOPE air quality modelling system (http://www.bsc.es/caliope) to simulate the O_3_ dynamics over the IP during the selected episode. CALIOPE is described elsewhere (Baldasano et al., 2008, 2011; Pay et al., 2010, 2012; and reference therein). The system consists of the HERMESv2.0 emission model (Guevara et al., 2013), the WRF-ARWv3.6 meteorological model (Skamarock and Klemp, 2008), the CMAQ v5.0.2 chemical transport model (Byun and Schere, 2006) and the BSC-DREAM8bv2 mineral dust model (Basart et al., 2012). CALIOPE first runs over Europe at a 12 × 12 km^2^ horizontal resolution (EU12 domain) and then over the IP at a 4 × 4 km^2^ resolution (IP4 domain) (Fig. S1 in the Supplement). In the present work, the system is configured with 38 sigma layers up to 50 hPa, both for WRF and CMAQ. The planetary boundary layer (PBL) is characterized by approximately 11 layers, and the bottom layer’s depth is ~ 39 m. The EU12 domain uses meteorological initial and boundary conditions from the final analyses provided by the National Centers of Environmental Prediction (FNL/NCEP) at a 0.5° × 0.5° resolution. The first 12 h of each meteorological run are treated as cold start, and the next 23 h are provided to the chemical transport model. Boundary conditions for reactive gases and aerosols come from the global MOZART-4/GEOS-5 model at 1.9° × 2.5° horizontal resolution (Emmons et al., 2010). CMAQ×uses the CB05 gas-phase mechanism with active chlorine chemistry, an updated toluene mechanism (CB05TUCL; Whitten et al., 2010; Sarwar et al., 2012) and the sixth-generation CMAQ aerosol mechanism including sea salt, aqueous/cloud chemistry and the ISORROPIA II thermodynamic equilibrium module (AERO6; Reff et al., 2009; Appel et al., 2013). Table S1 in the Supplement depicts the remaining CALIOPE configuration options.

For the IP4 domain, HERMESv2.0 estimates emissions for Spain with a temporal and spatial resolution of 1 h and up to 1 km × 1 km, according to the Selected Nomenclature for Air Pollution (SNAP), which are then aggregated to a 4 × 4 km^2^ resolution (Guevara et al., 2013). HERMESv2.0 is suitable for source apportionment studies thanks to its level of detail in the calculation of the emission fluxes by source (Guevara et al., 2014). The model is currently based on data from 2009, which was the closest year with updated information on local emission activities in HERMESv2.0 at the time that this work started. For neighbouring countries and international shipping activities, HERMESv2.0 uses the annual gridded national emission inventory provided by the European Monitoring and Evaluation Programme (EMEP) disaggregated to a 4 × 4 km^2^ resolution using a SNAP-sector-dependent spatial, temporal and speciation treatment (Ferreira et al., 2013).

HERMESv2.0 integrates the Model of Emissions of Gas and Aerosols from Nature (MEGANv2.0.4; Guenther et al., 2006) to estimate VOCs and NO_*x*_ emissions from vegetation, which play a major role in O_3_ photochemistry, using temperature and solar radiation from the WRF model. Note that we configured MEGAN to compute VOC emissions from cultivated crops; the agriculture emission module in HERMESv2.0 estimates the VOCs from manure management and field burning of agricultural residues. In this study, we have updated MEGANv2.0.4 with emission factors from MEGANv2.1 (http://lar.wsu.edu/megan/guides.html, last access: 4 April 2019). In Sect. 2 of the Supplement, we provide a comparison with measurements from the DAURE campaign (Pandolfi et al., 2014) that shows the reasonably good behaviour of our modelled isoprene.

**Figure 1.**
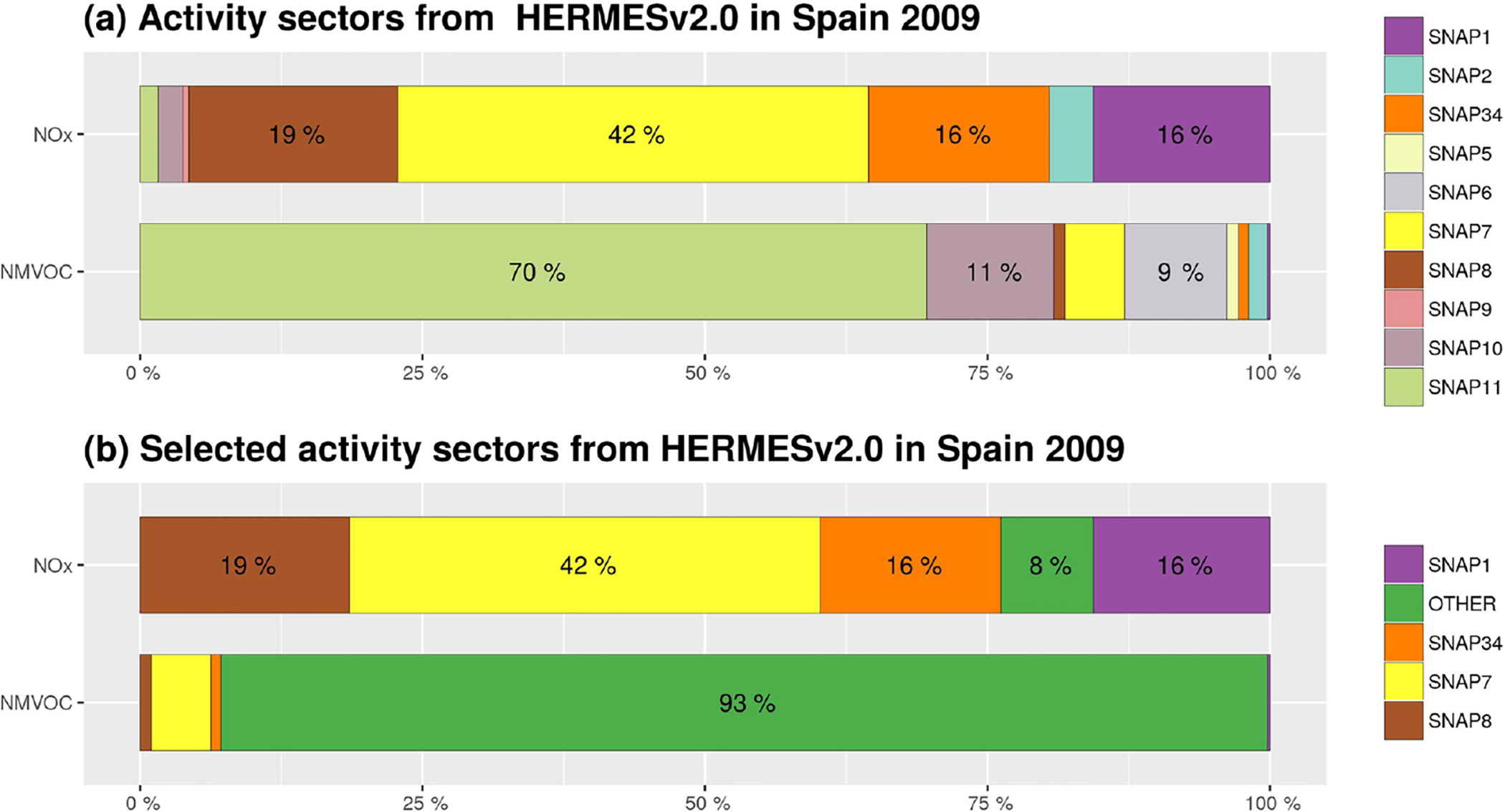
Percentage of the contribution of emissions to total annual emissions by SNAP sector calculated by HERMES for Spain 2009 **(a)** and for the selected SNAP sector accounting for more than 90 % of NO_*x*_ total emission to be tracked with ISAM **(b)**. “OTHER” compiles the SNAP categories 2 (residential combustion), 5 (fugitive emissions from fuels), 6 (solvent use), 9 (waste management), 10 (agriculture) and 11 (other sources). (NMVOC refers to non-methane volatile organic compounds.)

Urban VOC emissions could be a relevant source of O_3_. Over Spanish urban areas, HERMESv2.0 estimates VOC emissions from road transport and the use of solvents (Fig. 1) following bottom-up approaches (Guevara et al., 2013). However, uncertainties in the estimation of urban VOC emission inventories, as stated recently by several works (Pan et al., 2015; Liu et al., 2017; McDonald et al., 2018; Lewis, 2018) makes the urban VOC contribution to tropospheric O_3_ uncertain. In order to overcome this problem, continuous monitoring of urban VOCs should be performed in Spanish cities, following the example of other regions in which O_3_ is also a major problem such as Mexico City (Jaimes-Palomera et al., 2016). In addition, the use of formaldehyde satellite observations to constrain urban VOC emissions could also be pointed out as a future task to improve the representativeness of urban emission inventories (Zhu et al., 2014).

### 2.2 Ozone source apportionment method

We applied ISAM to quantify contributions from different SNAP categories to the surface O_3_ over the IP. The ISAM O_3_ tagging method is a mass-balance technique that tags both O_3_ and its gas precursor emissions (NO_*x*_ and VOC) from each source sector within one simulation (Kwok et al., 2013, 2015). Each tagged species undertakes typical physico-chemical processes (advection, vertical mixing, deposition, emission and chemistry) without perturbing the actual conditions. The O_3_ rate of change for each tag in any grid cell is calculated as follows (Eq. 1):

(1) $$\frac{\text{d}C_{\text{tag}}}{\text{d}t}=P_{\text{tag}}-D\frac{C_{\text{tag}}}{\underset{\text{tag}}{\sum }C},$$

where *C*_tag_ represents the O_3_ concentration related to a tagged source of interest, *P*_tag_ is the chemical production rate of O_3_ formed by the precursors emitted for each tag and *D* is the total chemical destruction rate of O_3_ in this grid cell. Different ratios of NO_*x*_/VOC cause the formation of O_3_ in each grid cell, which is either controlled by NO_*x*_ - or VOC-limited conditions. ISAM uses the H_2_O_2_/HNO_3_ ratio to determine whether O_3_ is NO_*x*_ - or VOC-sensitive (above or below 0.35, respectively) (Zhang et al., 2009). The bulk O_3_ concentration in each model grid cell (*P*_bulk_) is equal to the sum of O_3_ tracers that were produced under either NO_*x*_ - or VOC-sensitive conditions (Eq. 2),

(2) $$P_{\text{bulk }}=\underset{\text{tag }}{\sum }P_{\text{tag }}=\underset{\text{tag }}{\sum }P_{\text{tag }}^{\text{N}}+\underset{\text{tag }}{\sum }P_{\text{tag }}^{\text{V}},$$

where ${\text{P}}_{\text{tag}}^{\text{N}}$ and ${\text{P}}_{\text{tag}}^{\text{V}}$ are the O_3_ produced under NO_*x*_ - and VOC-limited conditions, respectively, according to Eqs. (3) and (4):

(3) $$P_{\text{tag}}^{\text{N},\text{new}}=P_{\text{tag}}^{\text{N},\text{old}}+P_{\text{bulk }}^{\text{new}}\frac{\underset{x}{\sum }{\text{NO}}_{x},\text{tag}}{\underset{\text{tag}}{\sum }\underset{x}{\sum }{\text{NO}}_{x},\text{tag}},$$

(4) $$P_{\text{tag}}^{\text{V},\text{new}}=P_{\text{tag}}^{\text{V},\text{old}}+P_{\text{bulk }}^{\text{new }}\frac{\underset{y}{\sum }{\text{VOC}}_{y,\text{ tag }}\times {\text{MIR}}_{y}}{\underset{\text{tag}}{\sum }\underset{y}{\sum }{\text{VOC}}_{y,\text{ tag }}\times {\text{MIR}}_{y}}.$$

In Eqs. (3) and (4), NO_*x*,tag_ and VOC_*j*,tag_ are the respective concentrations of the *x* nitrogen and *y* VOC species in CB05 that participate in the photochemical O_3_ formation for each source sector tag and grid cell, and MIR_*y*_ is the maximum incremental reactivity factor of each *y* species of VOC emitted by each source-sector tag, corresponding to the O_3_ generating potential of each single VOC species (Carter, 1994).

### 2.3 Ozone tagged species

Table 1 summarizes the O_3_ tagged sources in the present study, and Fig. 1a depicts the HERMESv2.0 model estimates of the contribution from each SNAP category to the total emissions of O_3_ precursors in Spain. The largest NO_*x*_ sources are road transport (SNAP7, 42 %), non-road transport (SNAP8, 19 %), manufacturing industries (SNAP34, 16 %) and energy production (SNAP1, 16 %). VOCs are dominated by biogenic sources (SNAP11, 70 %) and to a lesser extent by the agricultural sector (SNAP10, 11 %), solvent and other product uses (SNAP6, 9 %), and road transport (SNAP7, < 7 %). The selected (tagged) SNAP categories in this study are the energy, industrial, road transport and non-road transport sectors (Fig. 1b), which account for 92 % of the total NO_*x*_ emissions in Spain. An additional tracer (OTHER) gathers the remaining emission categories that were not explicitly tracked (i.e. SNAP2, 5, 6, 9, 10 and 11).

In addition to the selected sources, we tracked the contributions of the chemical boundary conditions (BCON) and the initial conditions (ICON). BCON represents both the O_3_ directly transported through the IP4 domain boundaries and the formation of O_3_ resulting from precursors that are also transported through the boundaries. BCON O_3_ comes from the EU12 parent domain, which includes the O_3_ produced in Europe and the O_3_ transported at a global scale (both tropospheric and stratospheric O_3_) provided by the MOZART-4/GEOS model (Fig. S1 in the Supplement). In the following, we name BCON O_3_ as the imported O_3_ to the IP4 domain. Tagging the initial O_3_ allows for the quantification of the number of spin-up days to minimize the impact of model initialization. For the present run, we required 6 days of spin-up to set the contribution of initial conditions to less than 1 % of the net hourly O_3_ concentration over 95 % of the available O_3_ stations.

### 2.4 Evaluation method

We evaluate the simulated concentrations against air quality measurements from the Spanish monitoring stations that are part of the European Environment Information and Observation Network (EIONET; https://www.eionet.europa.eu/, last access: 4 April 2019). The EIONET network provides a relatively dense geographical coverage of the Spanish territory. During the 21–31 July episode, we used the measurements from 347 stations for O_3_ and 357 stations for NO_2_ with a temporal coverage above 85 % on an hourly basis. Figure S2 in the Supplement shows the distribution of the stations for O_3_ and NO_2_.

The evaluation based on discrete statistics includes the correlation coefficient (*r*), the mean bias (MB), the normalized mean bias (NMB) and the root mean square error (RMSE) (Appendix B). We used the “openair” package (Carslaw and Ropkins, 2012) for R (v3.3.2; R Core Team, 2016) to compute the statistics. We calculate statistics on an hourly basis for O_3_ and NO_2_, as well as for the regulatory MDA8 in the case of O_3_. The evaluation also takes the station type, following the categories established by the EEA into account (i.e. rural background, suburban background, urban background, industrial and traffic).

There are no direct evaluation methods for apportioned pollutants. Instead, we designed a diagnostic plot for source apportionment analysis at each individual receptor, including a time series of measured and observed O_3_ and NO_2_ concentrations as well as the simulated tagged sources. In addition, this plot includes the simulated wind speed and direction. These plots are helpful as they compare the modelled O_3_ and NO_2_ with the observations, while highlighting the sources and circulation patterns at least partly responsible for the model behaviour. This work will only discuss the source apportionment plots at key O_3_ receptor regions in detail, given the high number of stations (260) that simultaneously measure O_3_ and NO_2_.

Evaluation results are discussed together with the source apportionment results. On the one hand, the interpretation of the source apportionment results benefits from model evaluation. On the other hand, the source apportionment results support enhanced model evaluation as it allows for the identification of potential errors in emission estimates for specific sectors and/or in the chemical boundary conditions.

## 3 Results

### 3.1 Description of the ozone episode

**Figure 2.**
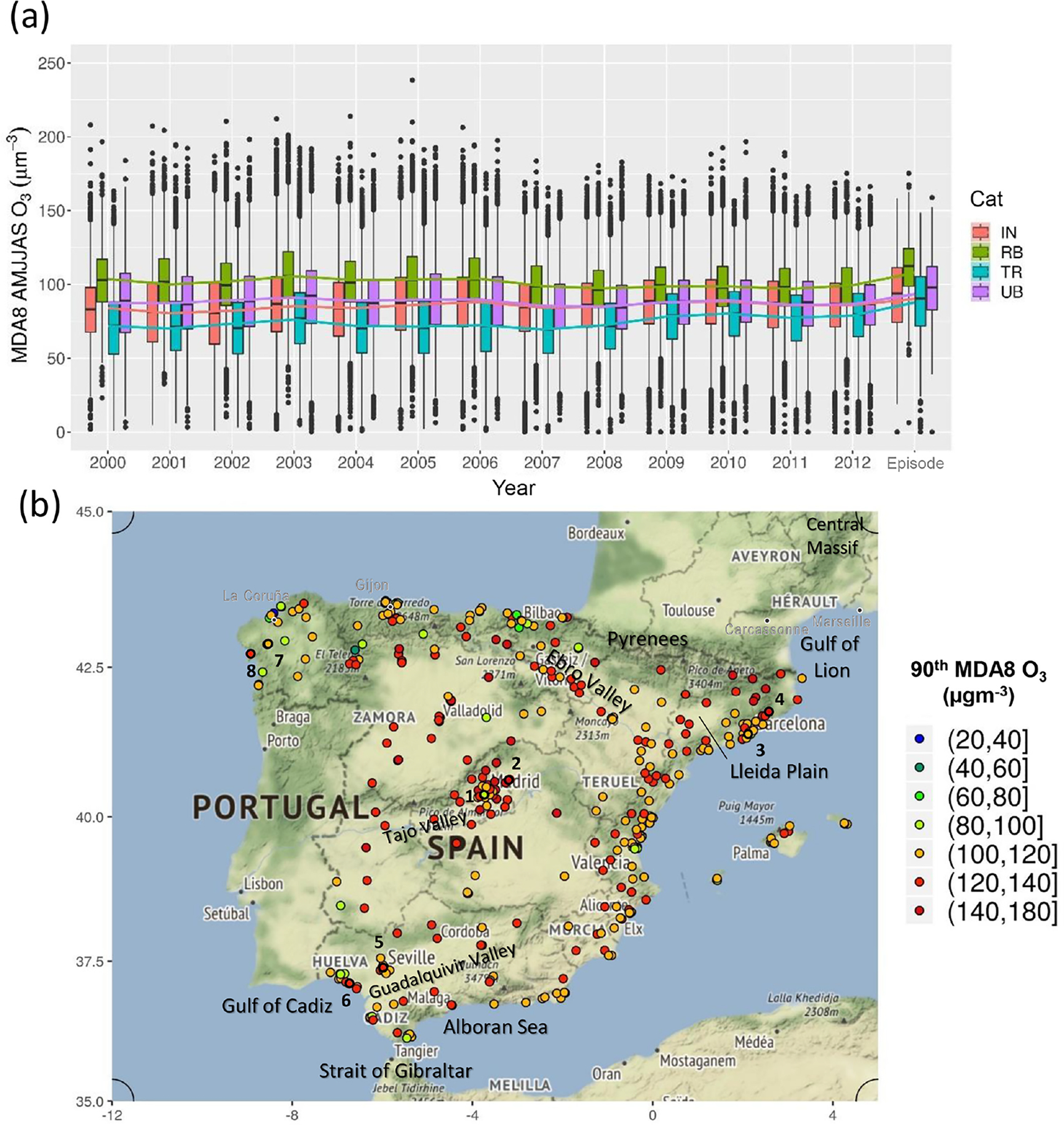
**(a)** Temporal distribution of the MDA8 O_3_ concentration during the extended summer (from April to September, AMJJAS) at the Spanish EIONET stations for the 2000–2012 period and the episode (from 21 to 31 July 2012) by station type: IN (industrial), RB (rural background), TR (traffic) and UB (urban background). **(b)** The 90th percentile of the MDA8 O_3_ concentration at the Spanish EIONET stations during the episode. Numbers indicate the stations cited in Sect. 3.4.

Our first estimation of the origin of peak O_3_ events in Spain focuses on the episode from 21 to 31 July 2012. Figure 2a illustrates the relevance of the episode showing the observed MDA8 O_3_ concentrations trends at the Spanish EIONET stations during the (extended) summers (i.e. from April to September) from 2000 to 2012, in addition to the concentrations recorded during the episode. Although the selected episode is not the most severe between 2000 and 2012 at a national scale, it comprises a period with high MDA8 O_3_ concentrations measured at rural background stations, (the 75th percentile of those values was above the target value) similar to the particularly severe summer of 2003 (Solberg et al., 2008).

This episode is also interesting because it was widespread and affected large areas of Europe (EEA, 2013). During this period alone, 33 % and 12 % of the total number of exceedances for the information threshold and the target value in 2012, respectively, were measured. The O_3_ regional context of the episode allows us to study the influence of the imported O_3_ to Spain.

The maps of the 90th percentile of the measured MDA8 O_3_ concentrations over Spain (Fig. 2b) show high concentration spots throughout the domain. The exceedances of the target value were found in the regions surrounding large urban areas (Madrid, Barcelona, Valencia and Seville) and along Spanish valleys (i.e. Ebro Valley and Guadalquivir Valley).

**Figure 3.**
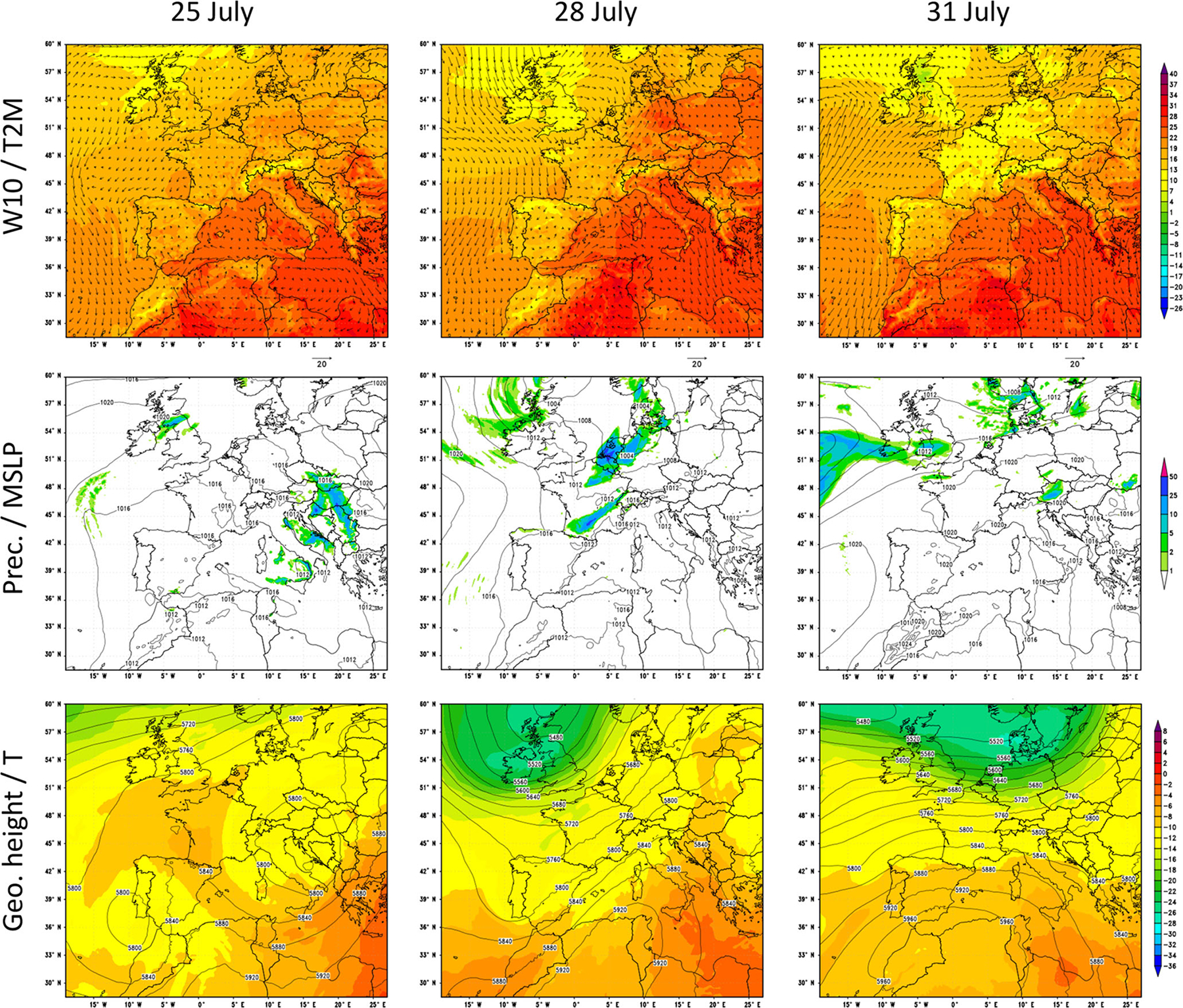
WRF-ARW meteorological fields at 06:00 UTC for 25, 28 and 31 July in the EU12 domain: 10 m wind speed (W10, ms^−1^), 2 m temperature (T2M, C), 6 h accumulated precipitation (Prec., mm), mean sea level pressure (MSLP, hPa), 500 hPa geopotential height in contours (Geo. height, m), and 500 hPa temperature in shaded colours (*T*, °C).

There were more than 100 exceedances of the O_3_ target value on most of the days during the episode, with relative maxima on 25, 28 and 31 July attributed to the change in the synoptic conditions (Fig. S3 in the Supplement). Figure 3 shows the meteorological patterns (2 m temperature, 10 m wind, precipitation, mean sea level pressure and geopotential height at 500 hPa) modelled by WRF-ARW during the 3 distinctive days over the outer EU12 domain.

Our characterization of the study period is based on the circulation type classification proposed in Valverde et al. (2014), who applied an objective synoptic classification method over the period from 1983 to 2012, specifically designed to study air quality dynamics over the IP. Stagnant conditions and northwestern advections are the most frequent summer synoptic circulation patterns over the IP, occurring on ~ 44 % of the days in a year (Jorba et al., 2004; Valverde et al., 2014). Stagnant conditions are characterized by reduced surface pressure gradients and weak synoptic winds, intense solar radiation, and the development of the Iberian thermal low (ITL). The ITL forces the convergence of surface winds from the coastal areas towards the central plateau enhancing sea breezes and mountain–valley winds and subsidence over the western Mediterranean Basin, as described by Millán et al. (1997, 2000) and Millán (2014). In contrast, northwestern advections (NWad) transport air masses from the Atlantic towards the north and west of the IP and they are characterized by atmospheric instability and intense ventilation. Periods of accumulation and venting of pollutants follow the same sequence of pressure ridging and troughing respectively, of the lower and middle troposphere of the IP during the warm season (Querol et al., 2017, 2018). According to the circulation type classification in Valverde et al. (2014), the selected episode started with the development of the ITL (21–25 July), followed by a NWad-venting period (26–29 July) and ended with the development of another ITL (30–31 July).

**Figure 4.**
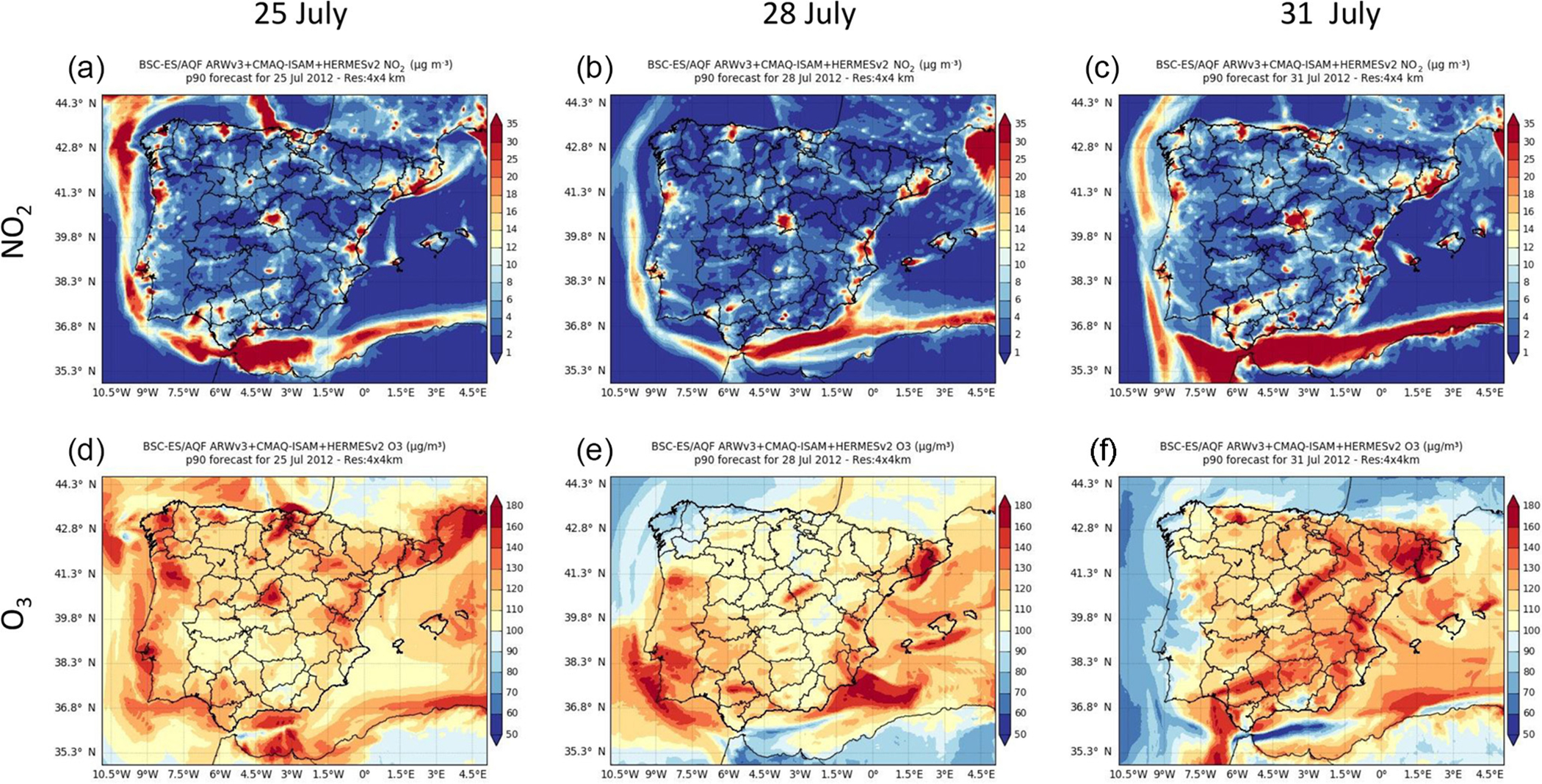
Ground-based concentration maps (in μg m^−3^) for NO_2_ **(a, b, c)** and O_3_ **(d, e, f)** corresponding to the 90th percentile of the average hourly concentrations on 25 **(a, d)**, 28 **(b, e)** and 31 **(c, f)** July 2012.

Figure 4 shows the 90th percentile (p90) of the simulated hourly O_3_ and NO_2_ concentrations corresponding to the 3 distinctive days with the relative maxima of exceedances. In the northern Spanish Mediterranean areas, intense O_3_ episodes often affect the plains and valleys located 60 km north of the Barcelona metropolitan area (BMA) in summer (Toll and Baldasano, 2000; Gonçalves et al., 2009; Valverde et al., 2016a; Querol et al., 2017). High NO_*x*_ concentrations from the BMA combined with high biogenic VOC levels are driven inland by mesoscale processes (sea breezes and mountain–valley winds). This happened on 31 July when the highest p90 of the hourly O_3_ concentrations (160–180 μg m^−3^) in Spain occurred over the north and northwest regions of the BMA. Occasionally, as occurred on 25 July, anticyclonic winds over the western Mediterranean Sea deflect the sea-breeze flow enriched with precursors from the BMA towards the Gulf of Lion where it reaches the highest p90 of the hourly O_3_ concentrations (160–180 μg m^−3^) in the IP Mediterranean region. Eastern Spanish Mediterranean areas show similar O_3_ dynamics, with inland regions depicting the highest O_3_ peaks (140–160 μg m^−3^) when stagnant conditions cover the central and eastern IP.

In the centre of the IP, intense O_3_ episodes occurred during the development of the ITL, where the affected area depends on the synoptic conditions (Querol et al., 2018). Under the absence of synoptic forcing (e.g. 25 July), the MMA had the highest p90 of the hourly O_3_ concentrations (~140–160 μg m^−3^). In contrast, when mountain-valley winds are reinforced with synoptic westerlies (e.g. 31 July; Fig. 3) the urban NO_*x*_ plume is channelled along the mountain ranges in Madrid towards the northeast and the highest p90 values of the hourly O_3_ concentrations are found along the valley (~ 140–160 μg m^−3^).

In the north and northeast of the IP, the p90 values of the hourly O_3_ concentrations show a significant increase when the blocking anticyclone over western Europe is combined with the development of the ITL (e.g. 25 July). The stagnant conditions favour the accumulation of O_3_ precursors around main cities and industrial areas and enhance the local O_3_ formation.

The NWad pattern (e.g. 28 July) significantly decreases the p90 of the hourly O_3_ concentrations in the centre and north of the IP. The northwesterly winds decrease the temperature and therefore the O_3_ formation. As a consequence, O_3_ levels are reduced in the plumes from the BMA and the MMA, although they are still significant in the latter. Overall, the p90 of the hourly O_3_ concentrations during the NWadv pattern was ~ 100 μg m^−3^ in most background areas. In contrast, during the ITL it was above 120 μg m^−3^.

### 3.2 Statistical evaluation

CALIOPE has been evaluated in detail elsewhere (Pay et al., 2014, and references therein). Furthermore, the system has been evaluated using the DELTA Tool (Thunis and Cuvelier, 2014) developed by the Forum for Air Quality Modelling in Europe to support and harmonize the model evaluation in the frame of the air quality directive. Valverde et al. (2016a, b) used DELTA Tool v4.0 and showed that CALIOPE accomplishes the quality objectives as defined in the air quality directive for 78 % of the NO_2_ and 91 % of the O_3_ monitoring stations during the summer 2012. Here, we evaluate the updated version of CALIOPE using ISAM to quantify the system’s ability to reproduce O_3_ and NO_2_ concentrations during the selected episode. Table 2 compiles the quartiles of the statistics calculated by station type.

The model slightly overestimates the average hourly and MDA8 O_3_ concentrations with MB values of +12 and +6 μg m^−3^, respectively. The *r* is above 0.6 at more than 50 % of the stations and above 0.7 at 25 % of them. The MB values for the average hourly and MDA8 O_3_ concentrations are lower at RB stations (±4 μg m^−3^) than at IN, TR and UB stations (between +6 and +16 μg m^−3^) at 50 % of the stations. As expected, the highest number of exceedances of the O_3_ target value was recorded at RB stations (260 exceedances) followed by IN stations (204 exceedances).

At RB stations, average hourly O_3_ is overestimated (+4 μg m^−3^) and MDA8 O_3_ is underestimated (−4 μg m^−3^), which indicates that nighttime O_3_ is overestimated. The nighttime overestimation is a common feature of CTMs and it is typically attributed to the underestimation of the O_3_ titration by NO (Bessagnet et al., 2016; Sharma et al., 2017).

CALIOPE underestimates the average hourly NO_2_ concentrations with −7 μg m^−3^ at TR stations and −2 μg m^−3^ at RB stations. This partly explains the high overestimation of the average hourly and MDA8 O_3_ concentration at TR and UB stations, as well as the systematic overestimation of average hourly O_3_ concentration at nighttime (due to a lack of O_3_ titration by NO). The average hourly NO_2_ concentration at TR stations features the highest *r* (with 25 % of stations above 0.6), which proves the reasonably accurate representation of temporal emission in urban areas by the HERMESv2.0 model (Guevara et al., 2014; Baldasano et al., 2011). In contrast, the RMSE is highest at TR stations, which results from the underestimation of NO_2_ peaks during traffic rush hours. Underestimation of NO_2_ traffic peaks is a common problem in Eulerian mesoscale models (Pay et al., 2014), as emission heterogeneity is lost in the grid cell-averaging process, which is especially critical in urban areas. Next generation microscale models will potentially solve this problem (Lateb et al., 2016). Besides the dilution of the emission in the grid, meteorology may also play an important role in the low performance of NO_2_ and O_3_ in hotspot areas. Several inter-comparison studies (e.g. EURODELTA and AQMEII) agree on the limitations of models to simulate meteorological variables that affect the average hourly NO_2_ temporal variability, which controls model performance for O_3_ in high-NO_*x*_ environments and their downwind areas (Bessagnet et al., 2016).

**Figure 5.**
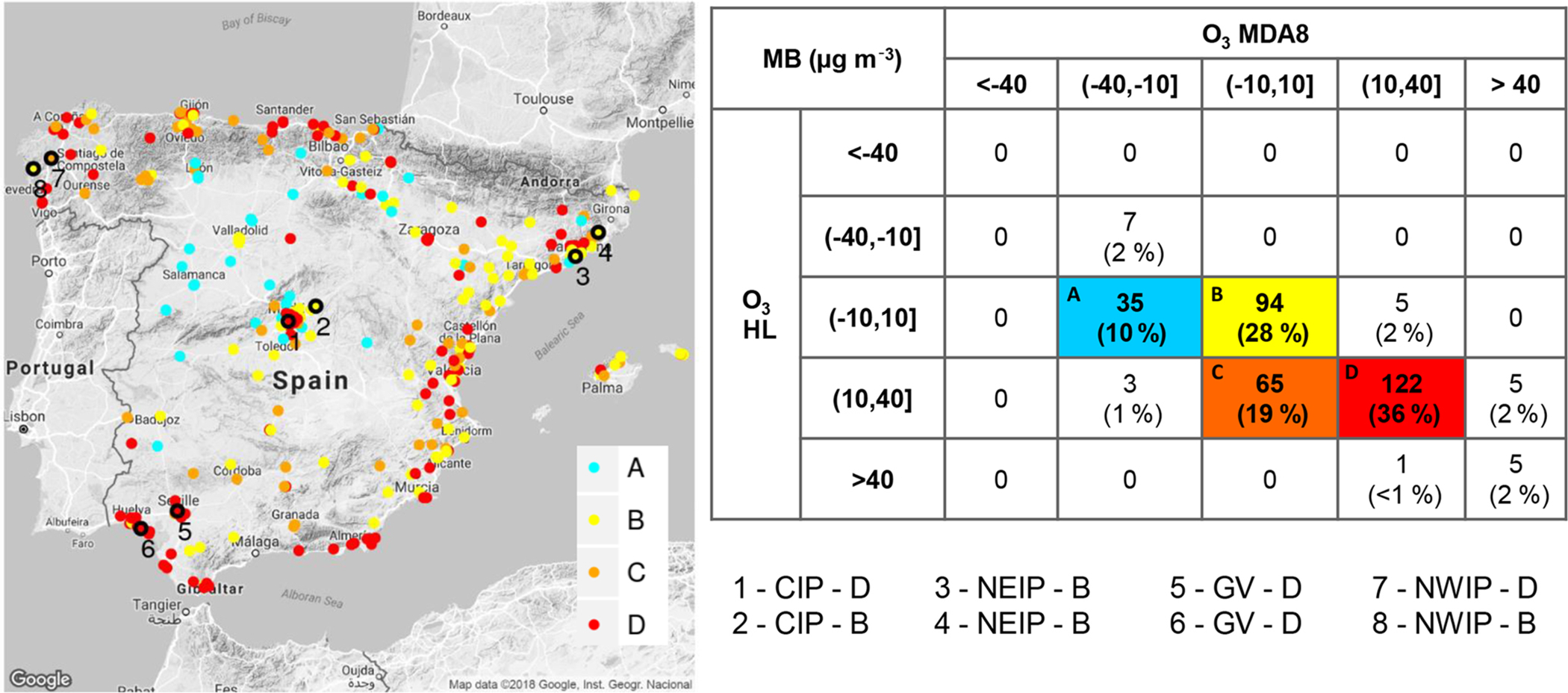
Air quality stations classified by both mean bias (MB, in μg m^−3^) for average hourly and MDA8 O_3_ at the Spanish EIONET stations and lumped by category (A, B, C and D). Numbered black circles indicate the stations under study in central IP (CIP; stations 1 and 2), northeastern IP (NEIP; station 3 and 4), Guadalquivir Valley (GV; stations 5 and 6) and northwestern IP (NWIP; stations 7 and 8).

Figure 5 classifies the average hourly and MDA8 O_3_ concentrations at the air quality stations into four MB categories that account for 93 % of the stations. The best performance for O_3_ (type B) is found at the 28 % of stations located in the areas surrounding the MMA, the BMA and most of the northern Mediterranean stations, which is consistent with the highest *r* (0.6 < *r* < 0.9) found in the centre and north of the IP (Fig. S4 in the Supplement). The highest O_3_ overestimations (type D) are present at the 36 % of stations that are mainly located in highly industrialized areas in Spain (Guadalquivir Valley, Strait of Gibraltar, Valencia) and inside the MMA. The next sections analyse the origin of these O_3_ biases using the source apportionment time series.

The comparison with previous CALIOPE studies (Baldasano et al., 2011; Pay et al., 2014) indicates that *r* is in the same range for O_3_ (0.6–0.7) and NO_2_ (0.4–0.5) at individual stations; the same applies for the RMSE (15–29 and 10–20 μg O_3_ m^−3^). Modelled O_3_ shows better performance at traffic stations in large cities, as stations influenced by road transport emissions (i.e. high-NO_*x*_ environments) are better characterized with a more pronounced daily variability (Baldasano et al., 2011). At the European scale, several model inter-comparisons (Giornado et al., 2015; Bessagnet et al., 2016) have indicated that O_3_ concentrations in summer agree with the surface observations with *r* values between 0.5 and 0.6. NO_2_ hourly variability is underestimated overall due to uncertainties in the emission estimates, meteorological inputs and model resolution. These studies highlight the limitations of models with respect to simulating meteorological variables that affect the NO_2_ hourly variability, and therefore the model performance for O_3_ in high-NO_*x*_ environments and their downwind areas.

Section S4 in the Supplement discusses the meteorological evaluation results and their impact on pollutant concentrations. Not surprisingly, temperature shows the best behaviour when compared with observations (Table S2 in the Supplement). The modelled wind speed is overestimated, particularly during nighttime (Fig. S5 in the Supplement), coincident with low-level wind speed. The nighttime overestimation of wind is a source of error in modelled NO_2_ and O_3_ nighttime concentrations (Vautard et al., 2012; Bessagnet et al., 2016).

### 3.3 Source-sector ozone contributions during peak episodes

**Figure 6.**
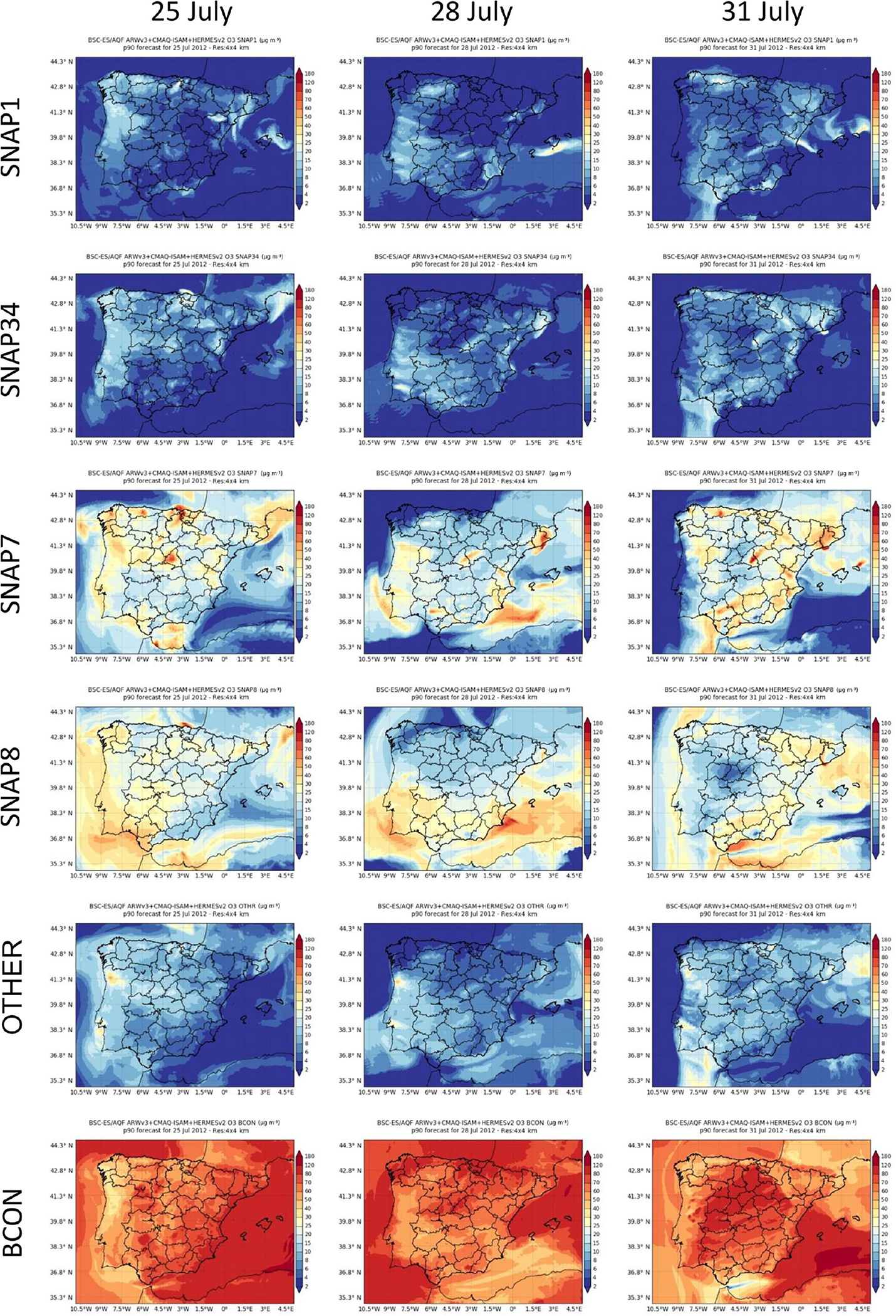
Tagged O_3_ concentrations (in μg m^−3^) corresponding to the 90th percentile (p90) of the average hourly concentrations: SNAP1, SNAP34, SNAP7, SNAP8, OTHER and BCON for 25 July (first column), 28 July (second column) and 31 July (third column) in 2012.

Figure 6 shows the p90 of the average hourly O_3_ concentration over the IP tagged by source type (Table 1) for different days (25, 28 and 31 July). (Fig. S6 in the Supplement shows similar plots for NO_2_.) The imported O_3_ is by far the largest contributor showing a p90 ranging from 70 to 120 μg m^−3^ in the east/north/centre of the IP on 25/28/31 July, respectively. The imported O_3_ enters the study domain through the IP4 domain boundaries and it can only be transported, scavenged, deposited or depleted by O_3_ precursors. Therefore, areas with low imported O_3_ concentrations (< 50 μg m^−3^) are good indicators of (1) the accumulation of specific O_3_ precursors that deplete imported O_3_, and (2) the subsequent O_3_ photochemical production that occur mostly under stagnant conditions and around the largest industrial/urban areas. The p90 of the hourly imported O_3_ concentration shows the lowest values under two different conditions and in regions. On 25 July in the northwestern IP and Portugal, stagnant conditions allow the accumulation of pollutants that titrate the imported O_3_ concentrations down to 30–70 μg m^−3^. At the same time O_3_ is locally produced downwind of major northern cities due to traffic emissions (La Coruña, Gijón, Bilbao) (60–120 μg m^−3^), shipping activities (up to 40 μg m^−3^) and the generation of energy and industrial processes (10– 20 μg m^−3^). On 31 July in the northeast of the IP, the pollutants transported from the Gulf of Lion and Catalonia towards the Mediterranean act as a sink of imported O_3_ reducing its concentration down to 60 μg m^−3^. As a result, there is local O_3_ formation up to 120–160 μg m^−3^ along the Ebro Valley and the Lleida Plain. In a source attribution study over northern Portugal, Borrego et al. (2016) also found a reduction of imported O_3_ and subsequent O_3_ formation by local sources under similar meteorological conditions.

Following imported O_3_, the largest contributor to O_3_ is the road transport sector. Downwind of major urban areas in Spain (i.e. Madrid, Barcelona, Bilbao, Seville and Valencia), on-road traffic contributed as much as 60–120 μg m^−3^ to the p90 of the hourly O_3_ concentrations, and affected different areas depending on the synoptic/mesoscale regimes (Fig. 6). In the north of the BMA, the p90 of the hourly O_3_ concentration from the road transport sector reaches its maximum when stagnant conditions affect the centre and eastern IP (e.g. 31 July). As noted above, mesoscale winds carry traffic O_3_ precursors from the BMA inland, channelled by north-south valleys towards the intra-mountain plain in the north. Over the MMA, the p90 of the hourly O_3_ concentration from the road transport sector showed a maximum when the ITL was combined with the synoptic westerlies (e.g. 31 July), carrying high O_3_ as far as the Ebro Valley, as shown in Fig. 4.

Regarding the contribution from the non-road transport sector, the Atlantic regions of the IP show the highest p90 of the average hourly O_3_ concentration (25–40 μg m^−3^) on 25 July. The stagnant conditions favoured the accumulation of precursors from the Atlantic shipping route and the formation of O_3_ within the region. The Spanish Mediterranean region shows the highest p90 of the average hourly O_3_ concentrations from the non-road transport sector close to the south-eastern coasts of the IP (~ 180 μg m^−3^) when the westerlies in the Strait of Gibraltar inject precursors from international shipping into the Mediterranean Basin (e.g. 28 and 31 July). Note that during days with high p90 of the average hourly O_3_ concentration from non-road transport, the imported O_3_ concentration shows the lowest p90 due to the NO titration effect over emission areas.

The elevated point source emission sectors (i.e. energy and industry) contributed less to O_3_ than the traffic sector, but their contributions were significant reaching 15–25 μg m^−3^ of the p90 of the average hourly O_3_ concentrations (Fig. 6). The north and northeast of the IP, the Mediterranean coast and the Guadalquivir Valley are the most affected regions under stagnant conditions.

The contribution of the remaining sectors (OTHER) to the p90 of the average hourly O_3_ concentrations was similar to that of the elevated point sources (15–25 μg m^−3^), but it reached up to 30 μg m^−3^ in areas downwind of Oporto and Lisbon (Fig. 6). OTHER includes the formation of O_3_ from the remaining anthropogenic and biogenic sources (accounting for less than 8 % of total NO_*x*_ emissions, but 93 % of total VOC). The high OTHER concentration around the biggest cities in Portugal may be related to precursors emitted by the residential sector (SNAP2 and SNAP9) and biogenic emissions, as found in other source apportionment studies over Portugal (Borrego et al., 2016; Karamchandani et al., 2017).

### 3.4 Regionalization of source-sector contributions

**Figure 7.**
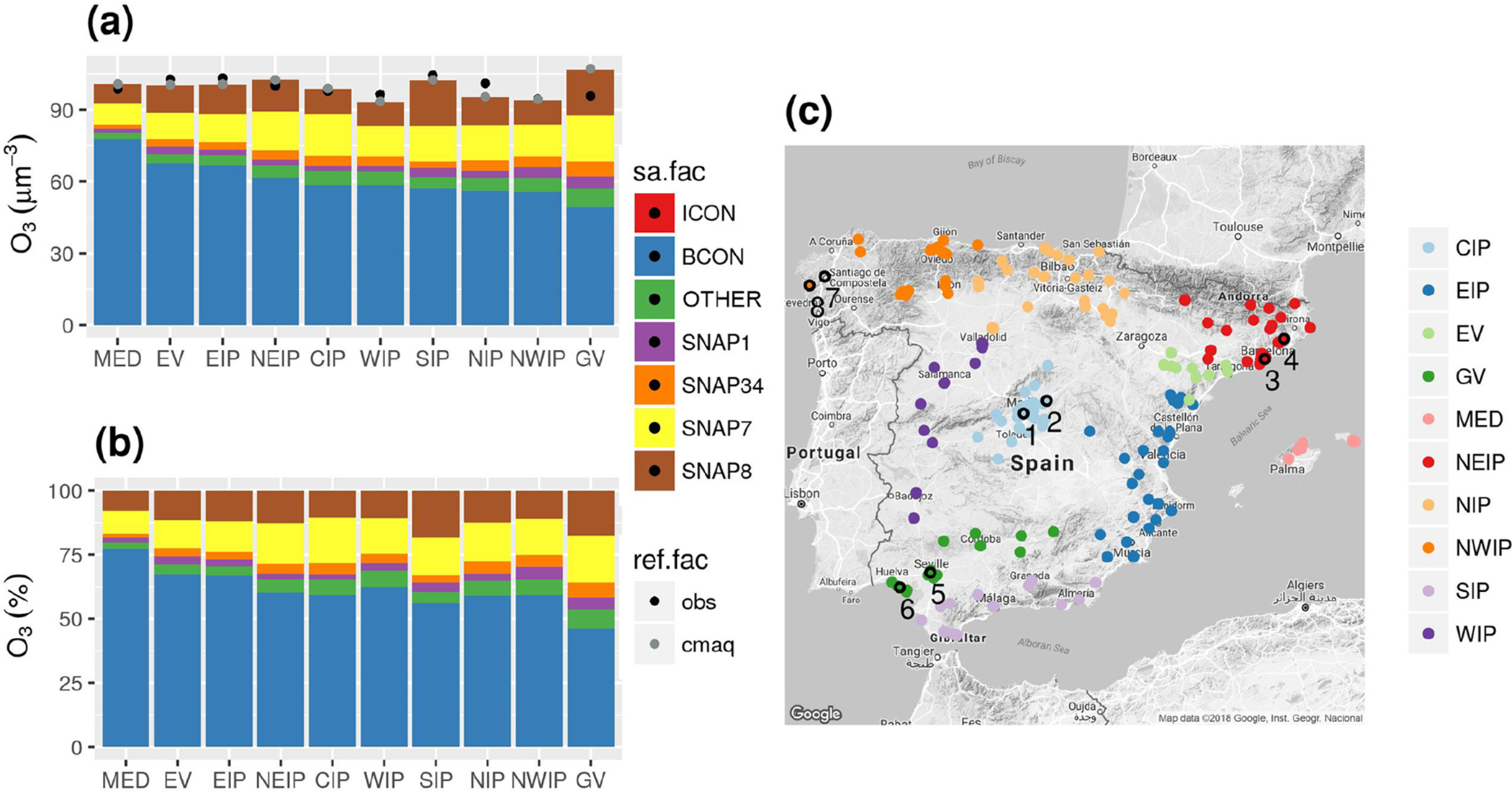
Daily mean contribution in μg m^−3^ **(a)** and in percentage **(b)** of tagged sources of O_3_ during exceedances of the observed 120 μg m^−3^ for MDA8 O_3_ averaged by the identified receptor regions **(c)**. Black and grey dots represent observed and modelled daily mean concentrations during exceedances of 120 μg m^−3^ of the observed MDA8 O_3_. Regions correspond to the centre of the IP (CIP), the east of the IP (EIP), the Ebro Valley (EV), the Guadalquivir Valley (GV), the Mediterranean Sea (MED), the northeast of the IP (NEIP), the north of the IP (NIP), the northwest of the IP (NWIP), the south of the IP (SIP) and the west of the IP (WIP). Numbered black circles indicate the stations under study CI (1–2), NEIP (3–4), GV (5–6) and NWIP (7–8).

We have identified 10 O_3_ receptor regions with similar characteristics in terms of meteorological and geographical patterns, O_3_ dynamics and main source contributors (Figs. 4 and 6). The receptor regions defined in our work are consistent with Diéguez et al. (2014) and Querol et al. (2016), who proposed a similar regionalization based on observations from air quality stations. Figure 7c shows the location of the air quality stations belonging to each receptor region corresponding to the centre of the IP (CIP), the east of the IP (EIP), the Ebro Valley (EV), the Guadalquivir Valley (GV), the Mediterranean Sea (MED), the northeast of the IP (NEIP), the north of the IP (NIP), the northwest of the IP (NWIP), the south of the IP (SIP) and the west of the IP (WIP).

Figure 7a shows the absolute O_3_ contribution of each tagged source at air quality stations by region along with the modelled and observed daily mean concentration during exceedances of 120 μg m^−3^ of the observed MDA8 ozone. Note that differences between sectors are more evident when normalizing (Fig. 7b). (Table S3 in the Supplement compiles the numerical values of Fig. 7.) Figure 7 indicates that during exceedances of the MDA8 target value there is a good agreement (*r* = 0.79) between the sum of apportioned O_3_ and the observed concentrations over the receptor regions.

The MED region shows the highest imported O_3_ contribution (76 %) because it is relatively far from important anthropogenic NO_*x*_ +VOC sources in the IP. Under the ITL influence (25 and 31 July), MED received air masses enriched with on-road traffic precursors from southern France and the NEIP, which enhanced O_3_ production up to 7 %. Shipping emissions in the MED region contributed up to 8 % of the total O_3_.

After MED, there is a cluster of regions along the Spanish Mediterranean coast (i.e. NEIP, EV and EIP) that show imported O_3_ contributions between 60 % and 68 % of the daily mean O_3_ under exceedances. This is explained by their proximity to the eastern boundary and the frequent mesoscale phenomena enhancing the recirculation and accumulation of imported O_3_ along the Spanish Mediterranean coast. The contribution of road and non-road transport is similar (~ 11 %–16 %) because these regions have both important roads and maritime trade routes. Note that the SIP region, which is also located on the Spanish Mediterranean coast, shows a daily mean imported O_3_ concentration lower than other regions along the Spanish Mediterranean coast (~ 57 %) and the highest non-road transport contribution in Spain (19 %). The main sink of imported O_3_ are precursors resulting from dense shipping traffic through the Strait of Gibraltar, which have a substantial impact on the O_3_ production downwind (either in the Alboran Sea or the Gulf of Cadiz).

The regions including the largest metropolitan areas in Spain are the CIP (Madrid) and the NEIP (Barcelona). Both regions show an imported O_3_ contribution of ~60 % and a similar contribution from the road transport sector (18 % and 16 %, respectively). However, the NEIP shows a slightly higher contribution from non-road transport (13 vs. 10 %) due to the influence of international shipping near coastal areas.

The northern and northwestern regions of the IP (NIP and NWIP) had relatively lower imported O_3_ contributions (56 %–59 %). The contribution of non-road transport was ~10 %–12 %, slightly lower than in the Mediterranean coast, and that of road transport was also significant (~14% – 15 %). The contribution from the industrial sector was one of the highest in the country (~ 5 %) which was probably related to the influence of the large industrial facilities located in several areas of the north of Spain. The contribution from the energy sector in the NWIP region was the highest in Spain (~ 5 %) due to emissions from large coal-fired power plants located in the area.

The WIP had the lowest daily mean O_3_ concentrations during days exceeding the O_3_ target value (93.5 μg m^−3^) and a high imported O_3_ contribution (~ 63 %). NO_*x*_ emissions in the WIP region are moderate (Fig. 4), which could explain the low daily mean O_3_ concentration. There is a significant contribution from traffic (14 % for road transport and 11 % for non-road transport) and industrial and energetic sectors (7 %) to the daily mean O_3_ concentrations. These anthropogenic contributions suggest that O_3_ in the WIP may be produced by precursors transported from the surrounding cities (Porto, Lisbon and Madrid) and the highly industrialized areas in the NWIP and the NIP (Fig. 6).

The Guadalquivir Valley had the lowest imported O_3_ contribution in the IP (~ 46 %) and the highest daily O_3_ concentration during days of exceedance. The on-road traffic was the highest anthropogenic contributor to O_3_ (~18 %) due to the emissions from three major cities (Seville, Huelva and Cordoba). Although O_3_ in Huelva may be overestimated (as discussed later), shipping is the second most important contributor to O_3_ in the Guadalquivir Valley (~17 %) which is probably linked to the important fluvial transport along the river. (The Guadalquivir River is one of the most important routes for merchandise transport in Europe.) In fact, the non-road transport sector is the highest contributor (~17 %–19 %) in southern Spain, both in the Guadalquivir Valley and the SIP, which is also due to the dense maritime routes across the Strait of Gibraltar.

The following sections analyse the source apportionment results at regions with a high on-road traffic contribution (i.e. CIP and NEIP) and a high contribution from industry and energy production (i.e. NWIP and Guadalquivir Valley).

#### 3.4.1 The centre of the Iberian Peninsula

**Figure 8.**
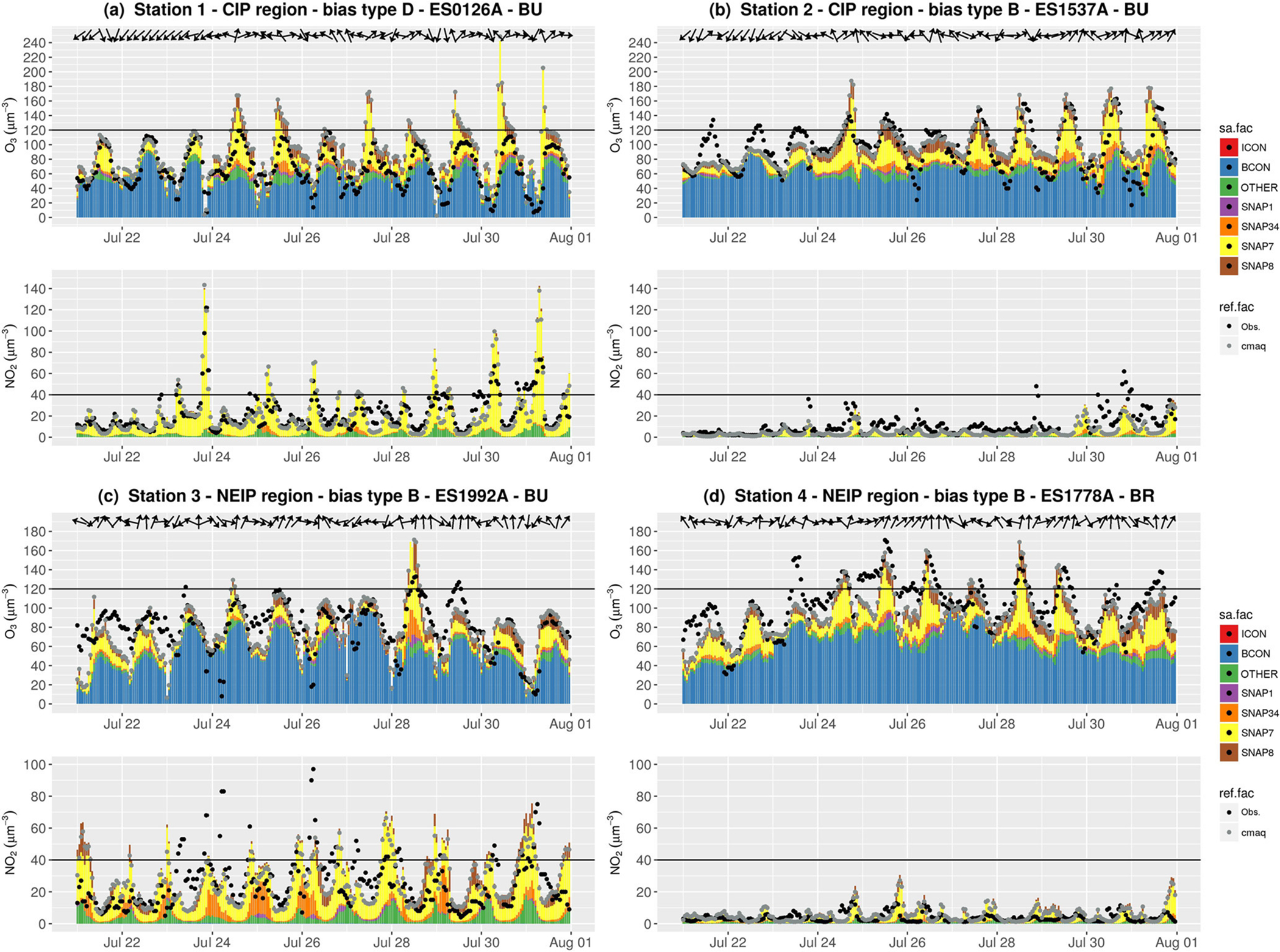
Source apportionment time series for O_3_ and NO_2_ concentrations (in μg m^−3^) in the episode at the selected stations in the centre of the IP (CIP) region **(a, b)**, and in the northeast of the IP (NEIP) **(c, d)**. Colour bars (sa.fac) indicate the O_3_ tags, and black and grey dots (ref.fac) indicate the respective observed and modelled concentrations. Black horizontal lines represent the O_3_ target value (120 μg m^−3^) and the NO_2_ limit value (40 μg m^−3^) as a reference. The locations of the stations are shown in Fig. 7 using the corresponding numbers.

Figure 8 shows the source apportionment time series of the average hourly O_3_ and NO_2_ concentrations at two stations, an urban station in Madrid (station 1 in Fig. 8a), and another station located in Guadalajara (station 2 in Fig. 8b), which is a medium size city affected by Madrid’s urban plume. At the urban station, the model reproduces the O_3_ traffic cycle (*r* = 0.66 and MB = 22.5 μg m^−3^) featuring the typical low O_3_ concentrations (< 40 μg m^−3^) in the early morning and in the afternoon due to O_3_ titration (Fig. 8a). However, O_3_ was overestimated (MB type D) during daytime peaks due to the overestimation of the NO_2_ morning peaks during stagnant conditions, coincident with the highest road transport contribution for both pollutants. The results point towards a poor representation of the meteorological condition in the city during stagnant conditions as shown in the meteorological evaluation (Sect. 4 in the Supplement).

At the urban station downwind (Fig. 8b), modelled O_3_ is positively biased (MB type B) due to the underestimation of NO_2_. Note that the uncertainty in NO_2_ traffic emissions in medium-sized cities is larger than in the largest urban areas (i.e. Madrid and Barcelona) because data are generally unavailable and emissions are estimated based on population density (Baldasano et al., 2011).

The imported O_3_ is the main contributor at both stations, but O_3_ formation due to traffic increases significantly during peaks at both stations. The highest O_3_ concentrations (~ 160 μg m^−3^) are modelled when westerly winds channelled along the Tajo Valley carry the polluted air masses in a northeasterly direction. This results in an O_3_ contribution of ~ 70 μg m^−3^ from the road transport sector in downwind areas (see wind vectors in Fig. 8b on 28 and 31 July). The O_3_ contribution from the industrial sector (whose precursors could come from facilities in the south of the MMA, Fig. 4) reinforces the O_3_ peaks up to ~ 10 μg m^−3^, whereas the contribution from non-road transport systematically increases the background O_3_ concentration by ~ 15 μg m^−3^. The O_3_ contribution from non-road transport in this region may arise mainly from Madrid’s airports and the agricultural machinery operating in the surrounding rural areas.

#### 3.4.2 The northeast of the Iberian Peninsula

Figure 8c and d show the source apportionment time series at two stations in the NEIP, an urban station in Barcelona (station 3) and a remote rural downwind area (station 4). Not surprisingly, at the urban station, NO_2_ levels of up to 100 μg m^−3^ affect O_3_ concentrations by titration during traffic peaks. In contrast, the rural station downwind depicts a higher O_3_ and lower NO_2_ concentration than the urban station. Absolute O_3_ biases at both stations are ~ 10 μg m^−3^ (MB type B).

O_3_ mostly results from import and from the NO titration effect due to local road transport and industrial sources (Fig. 8c). However, the O_3_ diurnal cycle in the urban areas of the NEIP is less marked than in the CIP due to the persistently high O_3_ concentration at night (~ 60 μg m^−3^). The breezes and mountain-valley winds contribute to the accumulation and recirculation of pollutants in this region.

At the rural station, modelled O_3_ peaks (> 120 μg m^−3^) are in a good agreement with observations (Fig. 8d), which suggests that the model generally reproduces the main transport paths, photochemical processes and relative contributions from different sources. Imported O_3_ is one of the main contributors to ground-level O_3_ (from 40 to 100 μg m^−3^), but during peaks the on-road traffic contribution sharply increases up to 80 μg m^−3^.

The O_3_ concentration from the road transport sector arriving at rural areas in the NEIP, mainly originates from Barcelona and its surroundings as a result of the afternoon sea breezes (see wind vectors in Fig. 8c and d). However, under specific meteorological patterns these winds also carry precursors from other cities located in the northwestern Mediterranean Basin. Other authors (Gangoiti et al., 2001) have hypothesized that the high O_3_ concentration in the western Mediterranean Basin is influenced by transport from France via the Carcassonne gap. The present experiment cannot quantify the contribution of French cities to the O_3_ concentration over the NEIP, but future studies could explicitly tag the emission from the French regions.

#### 3.4.3 Guadalquivir Valley

**Figure 9.**
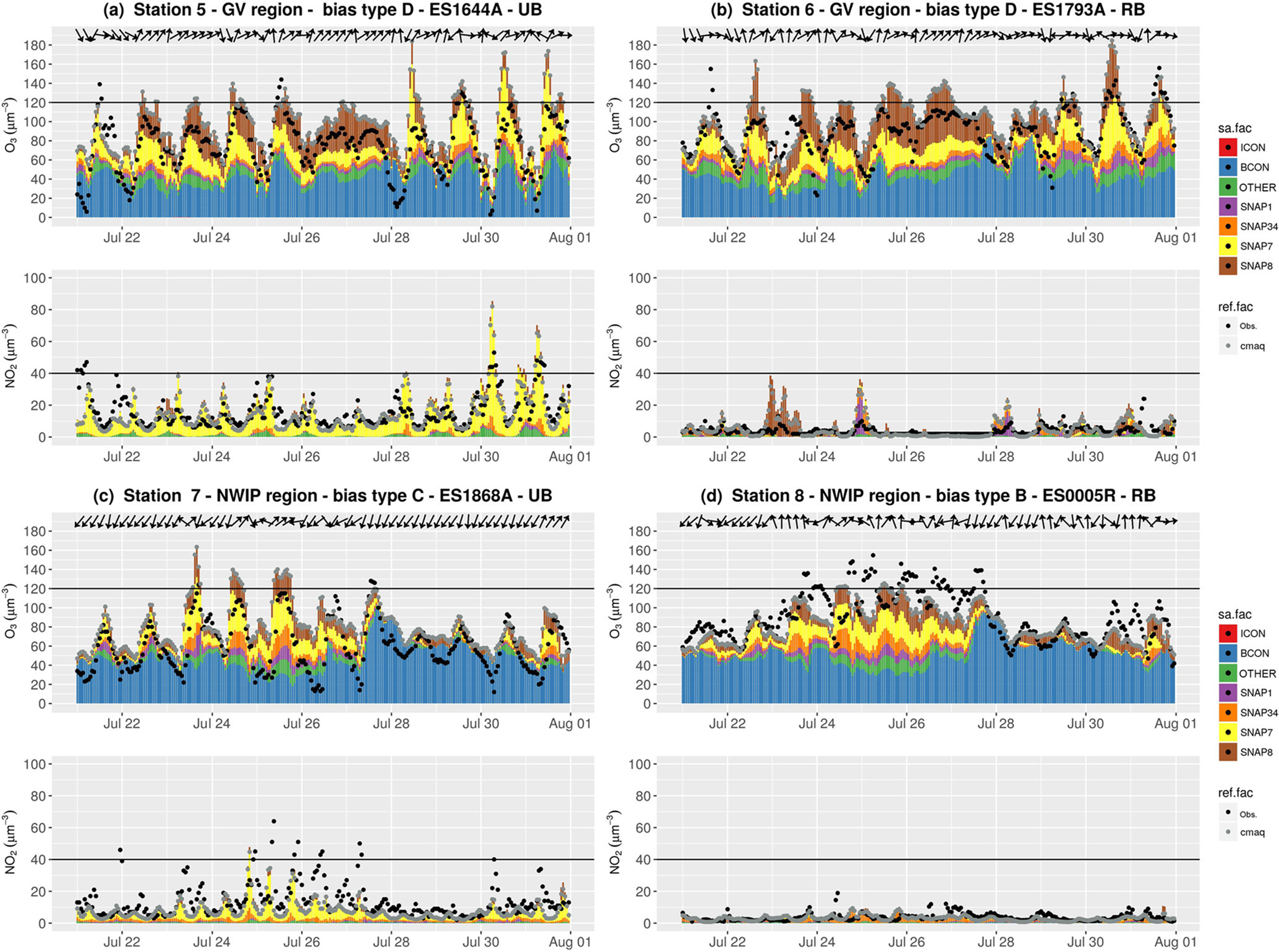
Source apportionment time series for O_3_ and NO_2_ concentrations (in μg m^−3^) in the episode at the selected stations in the Guadalquivir Valley (GV) **(a, b)**, and in the northwest of the IP (NWIP) **(c, d)**. Colour bars (sa.fac) indicate the O_3_ tags, and black and grey dots (ref.fac) indicate the respective observed and modelled concentrations. Black horizontal lines represent the O_3_ target value (120 μg m^−3^) and the NO_2_ limit value (40 μg m^−3^) as a reference. The locations of the stations are shown in Fig. 7 using the corresponding numbers.

We have selected two stations along the Guadalquivir Valley, one in the urban area of Seville (station 5 in Fig. 9a), and one in a rural coastal area (station 6 in Fig. 9b). The contribution of non-road transport is due the influence of one of the largest Spanish harbours. The contribution of the energy sector to the O_3_ concentration is also noticed (e.g. 2 July). As expected, the urban station (Fig. 9a) shows a high NO_2_ concentration dominated by on-road traffic. NO_*x*_ from traffic is the main sink of O_3_ in the city and the model reproduces the titration effect in agreement with observations (e.g. 28–31 July).

High O_3_ overestimations (10–30 μg m^−3^) at both stations (Fig. 9a and b) were detected during the 25–28 July period which correspond to intense and persistent southwesterly winds transporting air masses from the Atlantic Sea along the Guadalquivir Valley, as shown by the wind vectors in Fig. 9a and b. Although the model overestimates O_3_ concentrations, it reproduces the temporal variability.

Our results suggest that the non-road transport sector is a significant contributor along the Guadalquivir Valley during these days. The impact of shipping emission on O_3_ in the Guadalquivir Valley region is mainly evidenced by the relative high NO_*x*_ from ship exhaust (Fig. S3 in the Supplement). The NO_2_ time series at the coastal station (Fig. 9b) indicates that the model overestimates NO_2_ concentrations during hours when the NO_2_ contribution from non-road transport is highest. The unrealistic NO_2_ peaks from non-road transport suggest that shipping emissions are overestimated in the HERMESv2.0 model, which uses the EMEP gridded emission inventory at 50 km 50 km horizontal resolution to estimate shipping emissions×and spatially distributes them to the 4 × 4 km^2^ IP4 domain using the marine routes reported by Wang et al. (2008).

A recent review on the state-of-the-art of marine traffic emissions (Russo et al., 2018) indicates that STEAM appears as the most reliable and detailed emissions inventory, as it is based on Automatic Identification System data and specific vessel information, with a resolution of 2.5 × 2.5 km^2^ (Jalkanen et al., 2016). A comparative analysis×indicates that EMEP gridded inventories are overestimated, in particular over hotspots on the Mediterranean shipping routes, and underestimated on secondary routes. This factor, added to the 15 % decrease of NO_*x*_ shipping emissions observed in Europe between 2009 (HERMESv2.0 base year) and 2012 (EMEP CEIP, 2019) can at least partly explain the discrepancies observed.

#### 3.4.4 The northwest of the Iberian Peninsula

Figure 9c and d show the source apportionment time series at one urban (station 7) and one rural (station 8) background station in the NWIP. The urban station (Fig. 9c) located in Santiago de Compostela, a medium size city with ~100 000 inhabitants, shows a high NO concentration with 2 a dominant contribution from the road transport sector. Traffic NO_2_ is the main sink of urban O_3_ via titration. Because NO_2_ is underestimated, especially during stagnant conditions (24–27 July), O_3_ concentrations are overestimated (MB type C).

Despite the O_3_ biases during stagnant conditions, the modelled O_3_ concentration is in general agreement with observations at the rural background station (Fig. 9d). NO_2_ is likely to be underestimated due to missing traffic emissions. As previously noted, traffic emissions are poorly constrained in small and medium-sized cities, due to a lack of detailed information. There is also additional uncertainty in the precursors emitted from the large coal power plants and industries in the region (Valverde et al., 2016b). Our study uses emissions for 2009, and it has been estimated that between 2009 and 2012 energy production in coal-fired power plants increased from 13.1 % to 19.4 % (IEA, 2019). This implies an increase in NO_*x*_ emissions from the power industry sector of around 19.5 % (EMEP CEIP, 2019).

The time series show that the model reproduces the observed O_3_ variability reasonably well under different synoptic conditions. O_3_ reaches its highest concentrations (~ 100/150 μg m^−3^ in urban/rural areas) under stagnant conditions (24–27 July) when the contribution of anthropogenic sources from all activity sectors is highest (60 %–70 %). O_3_ concentrations decrease down to ~ 70 μg m^−3^ under northwest advective conditions (e.g. 28–30 July) when the imported O_3_ shows the highest contribution (80 %–90 %). Saavedra et al. (2012) found that stationary anticyclones over the NWIP play an important role in the occurrence of high O_3_ concentrations. Our results show that under these stagnant conditions O_3_ concentrations are largely due to in situ production (photochemistry) from on-road traffic, shipping, power plants and industry in almost the same proportion.

### 3.5 Imported ozone

Our results indicate that imported O_3_ represents the highest contribution to the ground-level O_3_ concentration in southwestern Europe. Imported O_3_ enters the IP4 domain through the boundaries; it includes the contribution of O_3_ from the EU12 domain, which in turn includes the contribution of hemispheric O_3_ from the MOZART-4 global model. The imported O_3_ contribution is as large as the background O_3_ regionally produced within the IP. Note that the small biases at rural background stations obtained in the evaluation section indicate an overall high performance of the modelled background O_3_ in the IP4 domain (Fig. S1 in the Supplement). Given the important implications and robustness of these results, we further analyse this contribution below. In particular, we aim to understand its high contribution within the IP, even far from the model domain boundaries.

**Figure 10.**
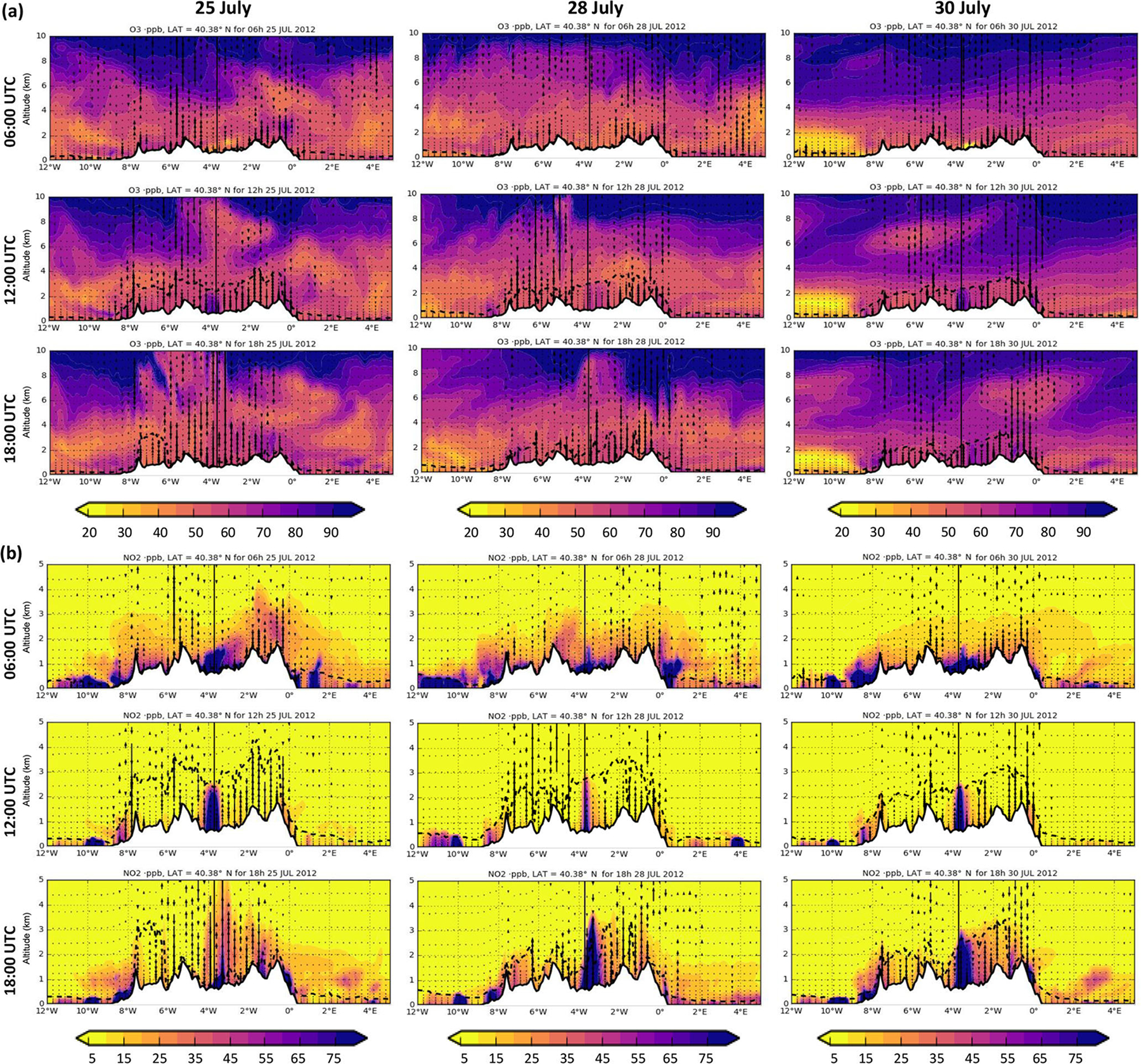
Cross section of modelled mixing ratios (in ppb) for O_3_ **(a)** and NO_2_ **(b)** at a constant latitude (latitude 40.38°, Madrid city) of the daily Iberian thermal low circulation, equivalent to the conceptual scheme of Millán et al. (1996) for 25 July=(first column), 28 July (second column) and 30 July (third column) at 06:00, 12:00 and 18:00 UTC. Dotted lines indicate the PBL height. Vertical arrows indicate the vertical wind. Up arrows depict positive winds. Note the different scales of the *y* axes between O_3_ and NO_2_.

Figure 10 shows the vertical cross-sections at 06:00, 12:00 and 18:00 UTC for O_3_ and NO_2_ at a constant latitude (40.38° N) on 25, 28 and 30 July. It helps to understand the vertical variability of both pollutants according to the PBL as schematized by Millán et al. (1996). The model predicts a pronounced O_3_ vertical gradient above a height of 4 km above sea level (a.s.l.), showing that O_3_ in the free troposphere is, to a large extent, imported to the IP4 domain (Safieddine et al., 2014). As expected, NO_2_ mixing ratios show a negative gradient with altitude as it is mainly emitted at the surface. In the morning, the sun starts to heat the ground up, producing convective thermals and forcing the growth of the mixing layer. At noon, the mixing height reaches its maximum: it is highest in the CIP (2–4 km) and decreases towards the coast (< 1 km) (Fig. 10). At the top of the mixing layer the O_3_-enriched air aloft is entrained into the mixing layer, mixing with O_3_ and other pollutants produced locally within the mixing layer. When the mixing height decreases, O_3_ is left in the free troposphere forming high O_3_ residual layers (Gangoiti et al., 2001) that contribute to the regional transport. Over the following days, these residual layers (composed of imported O_3_ and local O_3_ produced within the domain over previous days) can be entrained by fumigation into the mixing layer to reach the surface. This fumigation effect, previously described in the eastern USA (Zhang and Rao, 1999; Langford et al., 2015) and in the western Mediterranean Basin (Kalabokas et al., 2017; Querol et al., 2018), leads to a rapid increase in O_3_ concentrations at ground level. The accumulation and recirculation of air masses is intensified along the eastern Mediterranean coast (Millán et al., 1996, 2000; Gangoiti et al., 2001; Querol et al., 2017) by the action of the breezes and mountain–valley winds. Furthermore, the small deposition velocity of O_3_ over the sea and its high atmospheric lifetime in the free troposphere contributes to enrich the O_3_ background concentration (ca. several weeks, Monks et al., 2015; Seinfeld and Pandis, 2016).

The O_3_ fumigation effect was particularly intense from 30 to 31 July, when high O_3_ levels are found in the free troposphere compared with previous days (Fig. 10). The analysis of the O_3_ concentration map with the imported O_3_ contributions (Fig. 6) indicates that ground-based O_3_ is neither advected nor titrated; therefore, it can only result from vertical mixing. The high O_3_ mixing ratio in the free troposphere was mainly due to O_3_ advection entering the Atlantic boundary driven by westerlies (Fig. 3). We hypothesize that two events may have contributed to the increase of the O_3_ concentration by long-range transport: first, a low-pressure system in the British islands on 28 July could have transported significant amounts of O_3_ from the stratosphere to the free troposphere (Fig. S7 in the Supplement); second, O_3_ episodes generated in mid-July over the eastern USA predicted by the MOZART-4 model could have contributed to an increase in the O_3_ transported from North America to Europe by the action of the prevailing westerlies (Fig. S7 in the Supplement) associated with cyclonic systems along the “warm conveyor belt” (Pausata et al., 2012; Derwent et al., 2015).

## 4 Discussion and conclusions

Our study has provided a first estimation of the main sources responsible for high O_3_ concentrations in the western Mediterranean Basin during the 21–31 July 2012 period. We used the Integrated Source Apportionment Method (ISAM) within the CALIOPE system to estimate the contribution of the main anthropogenic activity sectors to peak O_3_ events in Spain compared with the imported O_3_. In addition, the use of ISAM has allowed an in-depth evaluation of the model.

The results demonstrate that the O_3_ problem over the western Mediterranean Basin is local, regional and hemispheric. Long-range transport of O_3_ from beyond the IP domain is the main contributor to the ground-level daily mean O_3_ concentration (~ 45 %) during peak episodes. The imported O_3_ contribution ranges from 40 % during O_3_ peaks to 80 % at night or during well-ventilated conditions. The absolute imported O_3_ is higher in the northeast of the IP than in the central IP due to the recirculation and accumulation of pollutants along the Mediterranean. The high imported O_3_ at the surface far away from the model boundaries is consistent with the high levels of O_3_ in the free troposphere (resulting from local/regional layering and accumulation, and continental/hemispheric transport) along with intense vertical mixing during the day.

Our results support the European Commission (EC, 2004) in pointing out that the effectiveness of abatement strategies for achieving compliance with the European air quality standards in southern Europe might be compromised by the long-range transport of O_3_. This is especially true in Mediterranean regions (i.e. NEIP, EV and EIP) where the contribution of imported O_3_ is particularly dominant (60 %–68 % of the daily mean O_3_ concentration) as a result of the accumulation and recirculation of pollutants over the Mediterranean Basin. In these areas, if the long-range transport of O_3_ is not reduced, the mean background level will not decrease, making it more vulnerable to exceedances of the O_3_ target values by enhanced local production under stagnant conditions.

During high O_3_ events, the imported O_3_ is added to the formation from local and regional anthropogenic sectors. Road transport is an important contributor to the O_3_ concentration in rural areas downwind of large cities in Spain; it contributed up to 16 %–18 % of the daily mean O_3_ concentration under exceedances of the target value for human health protection, and up to 70 μg m^−3^ on an hourly basis downwind of Barcelona and Madrid.

The non-road transport sector (including international shipping, airports and agricultural machinery) is as significant as road transport inland (10 %–19 % of the daily mean O_3_ concentration during the peaks). There is a high influence of international shipping (13 %), affecting the coastal areas in the Mediterranean and the south of the IP (along the Strait of Gibraltar) with contributions of up to ~ 20 and ~ 30 μg m^−3^, respectively. Although the non-road transport contribution was found to be overestimated in coastal areas in the south of the IP in the present experiment, it cannot be neglected; furthermore, actions controlling international shipping should be considered as important as those related to road transport, especially in regions with big harbours (e.g. Huelva and Barcelona). Dalsøren et al. (2010) indicated that the annual O_3_ concentration is increasing annually between 1 and 5 μg m^−3^ in areas impacted by shipping activities. Recent studies indicate that shipping emissions are projected to increase significantly due to increases in transportation demand and traffic. As the Strait of Gibraltar is the only shipping route connecting the Atlantic Ocean with the Mediterranean Basin, the regulation of these emissions is key in order to control O_3_ exceedances in Spain and the Mediterranean Basin. Shipping emissions can be regulated by each country within 400 km of coastlines, but policy-induced controls for offshore emissions are very dependent on the success of adopted and proposed regulations within the International Maritime Organization.

The energy and industrial sectors contribute ~ 6 %–11 % of the daily mean O_3_ concentration during the peaks and over all the receptor regions. As they are usually injected at high altitudes, their contribution extends way beyond their surroundings. The energy combustion sector (3 %) and industrial and non-industrial combustion sectors (3 %) have a mean contribution of 2–4 μg m^−3^, reaching to 4–6 μg m^−3^ in NO_*x*_-limited areas (i.e. the western IP). In highly industrialized regions (i.e. Guadalquivir Basin and northwestern IP), abatement strategies affecting all sectors at a regional scale could contribute to decrease the local formation of O_3_ as the regional/local anthropogenic contribution can be greater than 50 % over several days.

In the Barcelona metropolitan area the contribution from energy and industrial sectors to the NO_2_ concentrations can be in the same range as the contribution from road transport (~ 40 %–60 %, Fig. 8c). In contrast, in areas downwind of Barcelona the contribution from energy and industrial sectors to O_3_ concentrations is relatively low compared with the contribution from road transport (Fig. 8d). The different contributions to the O_3_ concentration might be related to the different reactivity of VOCs for O_3_ formation. Each VOC emission source emits a different mix of VOCs, which contributes differently to photochemical ozone formation. For example, in the UK, Derwent et al. (2007) showed a higher photochemical O_3_ creation potential for road transport emissions than for production processes and combustion. Future national policy actions to control the emissions of VOCs should tackle the sources that contribute more to photochemical O_3_ formation.

The remaining sectors (i.e. SNAP 2, 5, 6, 9 and 11; see Table 1), are the fourth main contributors to the daily mean O_3_ concentration during the days exceeding the target value (~ 2 %–8 %). Future work should tag biogenic sources as an individual sector as they are the main contributor to VOC emissions in Spain (i.e. ~ 70 % in 2009 according to the HERMESv2.0 model).

The air quality and meteorological evaluations indicate that uncertainties in our model are in the same range as the most recent inter-comparison studies using state-of-the-art air quality models. In addition, our model evaluation and the source apportionment results have allowed for a better understanding of the origin of model errors related to emission estimates. Our methodological choice was to use a detailed bottom-up emission inventory instead of a typical top-down regional emission inventory. Bottom-up emissions, estimated using source-specific emission factors and activity statistics, accurately characterize pollutant sources and allow for more realistic results to be obtained than those reported by top-down or regional emission inventories. To understand the impact of the use of 2009 data to study the year 2012, we revised the EMEP Centre on Emission Inventories and Projections (EMEP-CEIP), which collects and reviews the national emission inventories from parties to the Convention on Long-range Transboundary Air Pollution. Between 2009 and 2012, total NO_*x*_ and non-methane volatile organic compound (NMVOC) emissions in Spain decreased by −10.6 % and −10.7 %, respectively (EMEP CEIP, 2019). For NO_*x*_, around 80 % of this reduction is linked to a reduction of road transport emissions, whereas in the case of NMVOCs ~ 50 % of the reduction is due to a decrease in industrial emissions. For our modelling study, we consider these differences as small and acceptable (and as not creating any major inconsistency). The difference of 10 %–15 % in emissions for certain precursors between 2009 and 2012 is within the typically larger ranges of uncertainty in emission inventories.

Another relevant and uncertain source of O_3_ is the VOC emitted in urban areas. Future research works should be devoted to the continuous monitoring of urban VOC and take advantage of satellite observations to improve speciation and spatial variability of urban VOC emissions.

We have identified two sources of uncertainty in the estimation of the imported O_3_. First, it depends on both the performance of the CALIOPE system over EU12 and on MOZART-4 at a global scale. The small biases at rural background stations support an overall high performance baseline background O_3_ in the IP4 domain (Fig. S1 in the Supplement). Second, our set-up involves some uncertainty in the estimation of the imported contribution of O_3_. In reality, there is a fraction of the imported O_3_ that may have been generated within the IP4 domain before the period of simulation (including the spin-up). We have assumed that this fraction is negligible and future works should check the extent to which this assumption is correct.

For regulatory applications, further source apportionment studies should target not only emissions from activity sectors, but also the source regions where the emission abatement strategies should be applied. In addition, future studies should preferentially cover multiple summer periods in order to improve representativeness. We note that our results cannot predict whether emission abatement will have either a positive or a negative effect in O_3_ changes due to the non-linearity of the O_3_ generation process. Subsequent source sensitivity analyses tailoring the identified main contribution sources could predict how O_3_ will respond to reductions in precursor emissions, which are essential to define the most efficient O_3_ abatement strategies in the western Mediterranean Basin.

Overall, we find that the imported O_3_ is the largest input to the ground-level O_3_ concentration in the IP during the episode studied. However, during stagnant conditions, the emission from local anthropogenic activities in the IP control the O_3_ peaks in areas downwind of the main urban and industrial regions. Furthermore, ground-level O_3_ concentrations are strongly affected by vertical downward mixing of O_3_-rich layers in the free troposphere, which result from local/regional layering and accumulation, and continental/hemispheric transport. The importance of both imported and local contributions to the O_3_ peaks in the IP demonstrates the need for detailed quantification of both contributions to high O_3_ concentrations for local O_3_ management. Furthermore, the influence of local sources and topographical and meteorological conditions in the high O_3_ concentration indicate the importance of designing O_3_ abatement policies at the local scale.

This work has quantified the local and imported contributions to O_3_ during an episode in a particular area in southwestern Europe. In addition, we have provided a perspective regarding the potential use of source apportionment methods for regulatory studies in non-attainment regions. Further O_3_ source apportionment studies targeting other non-attainment regions in Europe are necessary prior to designing local mitigation measures that complement national and European-wide abatement efforts.

## Supplementary Material

Supplement1

## Figures and Tables

**Table 1.** Description of the O_3_ tagged sources in the present study.

ISAM tag*	Emission by SNAP category	Description
SNAP1	SNAP1	SNAP1: energy industry
SNAP34	SNAP34	SNAP34: manufacturing industries (combustion and processes)
SNAP7	SNAP7	SNAP7: road transport, exhaust and non-exhaust
SNAP8	SNAP8	SNAP8: non-road transport (international shipping, airport and agricultural machinery)
OTHER	SNAP2 + SNAP5 + SNAP6 + SNAP9 + SNAP10 + SNAP11	SNAP2: residential and commercial/institutional combustion; SNAP5: fugitive emissions from fuels; SNAP6: product use including solvents; SNAP9: waste management; SNAP10: agriculture; and SNAP11: other sinks
BCON	-	Chemical boundary conditions of the IP4 domain from the EU12 simulation which includes the contribution from Europe and the international contribution from MOZART-4. O_3_ external contribution
ICON	-	Initial chemical condition of the IP4 domain

* Each ISAM tag is applied to O_3_ and its precursor species in the CB05 (NO_*x*_ and VOCs). NO_*x*_ species contributing to O_3_ formation involve (9 species): NO, NO_2_, nitrogen trioxide (NO_3_), dinitrogen pentoxide (N_2_O_5_), nitrous acid (HONO), peroxyacyl nitrates (PAN), higher peroxyacyl nitrates (PANX), peroxynitric acid (PNA) and organic nitrates (NTR). VOC species contributing to O_3_ formation include (14 species): acetaldehyde (ALD2), higher aldehydes (ALDX), ethene (ETH), ethane (ETHA), ethanol (ETOH), formaldehyde (FORM), internal olefin (IOLE), isoprene (ISOP), methanol (MEOH), olefin (OLE), paraffin (PAR), monoterpene (TERP), toluene (TOL) and xylene (XYL).

**Table 2.** Statistics for average hourly O_3_, MDA8 O_3_ and average hourly NO_2_ concentrations in the episode as a function of the station type. Exceedances indicate the number of exceedances of the European air quality directive standards for hourly O_3_ (180 μg m^−3^), MDA8 O_3_ (120 μg m^−3^) and average hourly NO_2_ (200 μg m^−3^). *N* indicates the number of monitoring stations used in the statistical calculation. MO and MM depict the measured and modelled mean concentrations, respectively. Statistics are calculated by considering more than 75% of the hours in a day, as established by Directive 2008/50/EC. The statistics correspond to the 50th (25th, 75th) quantiles by station. Type indicates the station categories in the calculation of statistics: all of the stations (ALL), industrial (IN), traffic (TR), urban background (UB), suburban background (SB) and rural background (RB) stations.

Pollutant	Type	Exceedances: observed/modelled	*N*	MO (μgm^−3^)	MM (μgm^−3^)	MB (μg m^−3^)	NMB (%)	RMSE (μgm^−3^)	*r*
Hourly O_3_	ALL	26/216	348	77.3 (66.7, 86.5)	89.9 (83.5, 95.6)	12.6 (4.4, 20.2)	16.8(5.1,29.2)	26.7 (21.5,32.1)	0.65 (0.57, 0.72)
	IN	5/23	106	74.1 (62.2, 83.2)	82.2 (83.4, 94.5)	14.1 (4.5,21.5)	19.8(5.5,34.7)	26.8 (21.1,32.8)	0.66 (0.56, 0.73)
	TR	0/58	70	68.4 (57.1,76.7)	83.8 (74.4, 89.2)	15.9 (8,21.8)	23.4 (10.1,38.5)	28.8 (24.5, 33.9)	0.63 (0.54, 0.70)
	UB	0/78	56	74.8 (64.1, 80.0)	89.4 (81.4, 94.5)	15.6(7.8,21.3)	22.1 (10.2,31.1)	28.2 (24.5, 33.5)	0.65 (0.60, 0.70)
	SB	4/48	44	83.2 (78.1,87.2)	93.5 (88.2, 97.3)	10 (4.6, 14.6)	12.9 (5.4, 17.5)	25.1 (21.4, 29.3)	0.66 (0.60, 0.72)
	RB	17/9	66	91.2 (80.9,97.1)	96.2 (92.6, 99.6)	4.5 (−3.3, 14.7)	4.8 (−3.2, 17.2)	21.2 (18.3, 28.0)	0.67 (0.57, 0.74)
MDA8 O_3_	ALL	751/822	348	101 (91.0, 113.4)	106.2(101.8, 113.2)	5.7 (−3.7, 16.9)	5.5 (−3.3, 17.4)	17.9 (13.5, 25.4)	0.64 (0.39, 0.78)
	IN	204/187	106	96.7 (88.9, 109.5)	104.8 (99.4, 109.2)	5.7 (−2.9, 16.5)	5.7 (−2.9, 17.8)	17.0 (13.0, 24.5)	0.68 (0.48, 0.82)
	TR	62/145	70	92.7 (83.6, 99.9)	104.1 (98.3, 110.2)	13.3 (4.6, 23.8)	15.1 (4.6,27.2)	21.2 (15.2,31.2)	0.54 (0.27, 0.76)
	UB	86/174	56	100.0(89.3, 105.5)	107.6 (102.2, 122.8)	14.1 (2.1,21.7)	14.8(2.1,22.5)	22.6(15.6, 28)	0.59 (0.34, 0.77)
	SB	139/131	44	110.7 (99.8, 118.5)	110.0(105.5, 119.2)	3.1 (−6.2, 10.5)	2.9 (−5.4, 10.1)	16.9 (13.6, 22)	0.5 (0.31, 0.67)
	RB	260/185	66	113.1 (104.9, 119.9)	108.6(104.5, 113.2)	−3.7 (−11.1, 5.5)	−3.3 (−9.1, 5.1)	15 (11.8, 20.2)	0.72 (0.60, 0.84)
Hourly NO_2_	ALL	3/0	358	14.0 (8.4, 21.2)	9.9 (4.4, 16.2)	−4.1 (−8.4, −0.5)	−31.9 (−54.2, −4.9)	12.8 (8.2, 17.6)	0.43 (0.29, 0.55)
	IN	0/0	120	11.5 (7.9, 16.3)	8.8 (3.8, 14.7)	−3.0 (−6.6, 0.2)	−28.6 (−55.0, 1.1)	11.0(7.9, 15.6)	0.40 (0.26, 0.50)
	TR	3/0	95	22.9 (17.7, 28.7)	14.1 (8.7,21.6)	−7.4 (−13.9,−2.6)	−35.2 (−58.1, −14.3)	18.4 (14.1,23.1)	0.42 (0.28, 0.57)
	UB	0/0	63	17.0(13,21.2)	13.2 (7.8, 18.4)	−4.0 (−8, −0.3)	−26.9 (−47.4, −2)	14.1 (10.5, 17.3)	0.49 (0.36, 0.63)
	SB	0/0	33	12 (8.2, 14.6)	7.6 (4.8, 11.1)	−2.8 (−6.5, 0.8)	−34.4 (−48.6, 7.5)	6.4 (7.9, 13.6)	0.5 (0.41, 0.65)
	RB	0/0	41	4.3 (3.5, 9.3)	2.8 (1.6, 4.0)	−2.0 (−5.3, −0.3)	−49.5 (−72.3, −9.7)	4.4 (2.7, 7.1)	0.34 (0.24, 0.43)

## Appendix A

**Table A1.** Table 1a shows the acronyms used in the text.

BCON	Chemical boundary conditions to IP4, also referred to as the imported O_3_ contribution
BMA	Barcelona metropolitan area
CIP	Central Iberian Peninsula
CMAQ	Community Multiscale Air Quality model
EIP	Eastern Iberian Peninsula
EU12	European domain at 12 × 12 km^2^ horizontal resolution.
EV	Ebro Valley
GV	Guadalquivir Valley
IP	Iberian Peninsula
IP4	Iberian Peninsula domain at 4 × 4 km^2^ horizontal resolution.
ISAM	Integrated Source Apportionment Method
MDA8	Maximum daily 8 h average concentration
MMA	Madrid metropolitan area
MED	Mediterranean Sea
NEIP	Northeast of the Iberian Peninsula
NIP	North of the Iberian Peninsula
NWIP	Northwest of the Iberian Peninsula
SIP	South of the Iberian Peninsula
SNAP1	Emission sector on combustion in energy
SNAP34	Emission sector on combustion and processes in industry
SNAP7	Emission sector on road transport, exhaust and non-exhaust
SNAP8	Emission sector on non-road transport (international shipping, airport and agricultural machinery)
WIP	West of the Iberian Peninsula
WRF	Weather Research and Forecasting model

## Appendix B

Definition of the discrete statistics used in the evaluation: correlation coefficient (*r*, Eq. B1), mean bias (MB, Eq. B2), normalized mean bias (NMB, Eq. B3) and root mean squared error (RMSE, Eq. B4). Where *C*_m_(*x*, *t*) and *C*_o_(*x*,*t*) are the respective modelled and observed concentrations at a location (*x*) and time (*t*); *N* is the number of pairs of data. $\bar{C_{\text{m}}}$ and $\bar{C_{\text{o}}}$ are the modelled and observed mean concentrations over the whole period, respectively.

(B1) $$r=\frac{\overset{N}{\underset{i=1}{\sum }}\left(C_{\text{m}}(x,t)-\bar{C_{\text{m}}}\right)\left(C_{0}(x,t)-\bar{C_{0}}\right)}{\sqrt{\overset{N}{\underset{i=1}{\sum }}{\left(C_{\text{m}}(x,t)-\bar{C_{\text{m}}}\right)}^{2}}\sqrt{\overset{N}{\underset{i=1}{\sum }}{\left(C_{0}(x,t)-\bar{C_{0}}\right)}^{2}}},$$

(B2) $$\text{MB}=\frac{1}{N}\overset{N}{\underset{i=1}{\sum }}\left(C_{\text{m}}(x,t)-C_{0}(x,t)\right),$$

(B3) $$\text{NMB}=\frac{\overset{N}{\underset{i=1}{\sum }}\left(C_{\text{m}}(x,t)-C_{0}(x,t)\right)}{\overset{N}{\underset{i=1}{\sum }}\left(C_{\text{o}}(x,t)\right)}\times 100\;=\left(\frac{\bar{C_{\text{m}}}}{\bar{C_{0}}}-1\right)\times 100,$$

(B4) $$\text{RMSE}=\frac{1}{N}\sqrt{\overset{N}{\underset{i=1}{\sum }}{\left(C_{\text{m}}(x,t)-C_{\text{o}}(x,t)\right)}^{2}}.$$
