# Supplementary material for: Ozone source apportionment during peak summer events over southwestern Europe
Source: Atmos Chem Phys. Author manuscript; Available in PMC 2021 Jan 7. (PMC7788066; doi:10.5194/acp-19-5467-2019)
Supplement: Supplement1 [file NIHMS1528606-supplement-Supplement1.pdf]

Supplement of Atmos. Chem. Phys., 19, 5467–5494, 2019
https://doi.org/10.5194/acp-19-5467-2019-supplement
© Author(s) 2019. This work is distributed under
the Creative Commons Attribution 4.0 License.

Atmospheric Chemistry and Physics
Open Access

Supplement of

Ozone source apportionment during peak summer events over southwestern Europe

María Teresa Pay et al.

Correspondence to: María Teresa Pay (maria.pay@bsc.es)

The copyright of individual parts of the supplement might differ from the CC BY 4.0 License.

Contents

S1. Configuration of the CALIOPE system in this study 2
S2. Evaluation of biogenic emissions 3
S3. Number of exceedances during the episode 4
S4. Model evaluation 4
S5. Tagged NO_2 concentrations 8
S6. Regionalization of source-sector contributions 9
S7. Ozone global maps 10

S1. Configuration of the CALIOPE system in this study

Table S1. Detail set up of the CALIOPE system in the present modelling study

**Meteorological model**	**WRF-ARWv3.5.1**
Initial and boundary meteorological conditions	GFS-FNL (1.5ºx1.5º, 6h)
Meteorological spin-up period	12 hours
WRF mother domain 12-km resolution over Europe (nx, ny, nz)	480 x 400 x 38
WRF nested domain 4-km resolution over the Iberian Peninsula (nx, ny, nz)	399 x 399 x 38
WRF parametrizations	Boundary layer: YSU<br>Microphysics scheme: WSM5<br>Cumulus scheme: no cumulus parametrization<br>Land Surf physics: unified Noah land-surface model<br>Long Wave: RRTM<br>Short Wave: Dudhia<br>Surface Layer: Revised MM5 Monin-Obukhov scheme
**Emission model**	**HERMESv2**
Base year of the emission	2009
**Chemical Transport Model**	**CMAQv5.0.2**
Chemical mechanism	cb05-TUCL-aero6
Chemical boundary conditions	MOZART4-GEOS5 forecast (1.7º x 2.5º, 6h)
CTM spin-up period	6 days
ISAM tracked species	O_3, NO_x, NMVOC
Mother domain 12-km resolution over Europe (nx, ny, nz)	478 x 398 x 15
Nested domain 4-km resolution over the Iberian Peninsula (nx, ny, nz)	397 x 397 x 15
CMAQ parametrizations	Horizontal advection scheme: Yamartino mass-conserving<br>Vertical advection scheme: Piecewise Parabolic Method (PPM)<br>Vertical diffusion: Asymmetric Convective Model v2 (ACM2) Eddy diffusivity approach<br>Dry deposition routine: Models-3 + Cl species

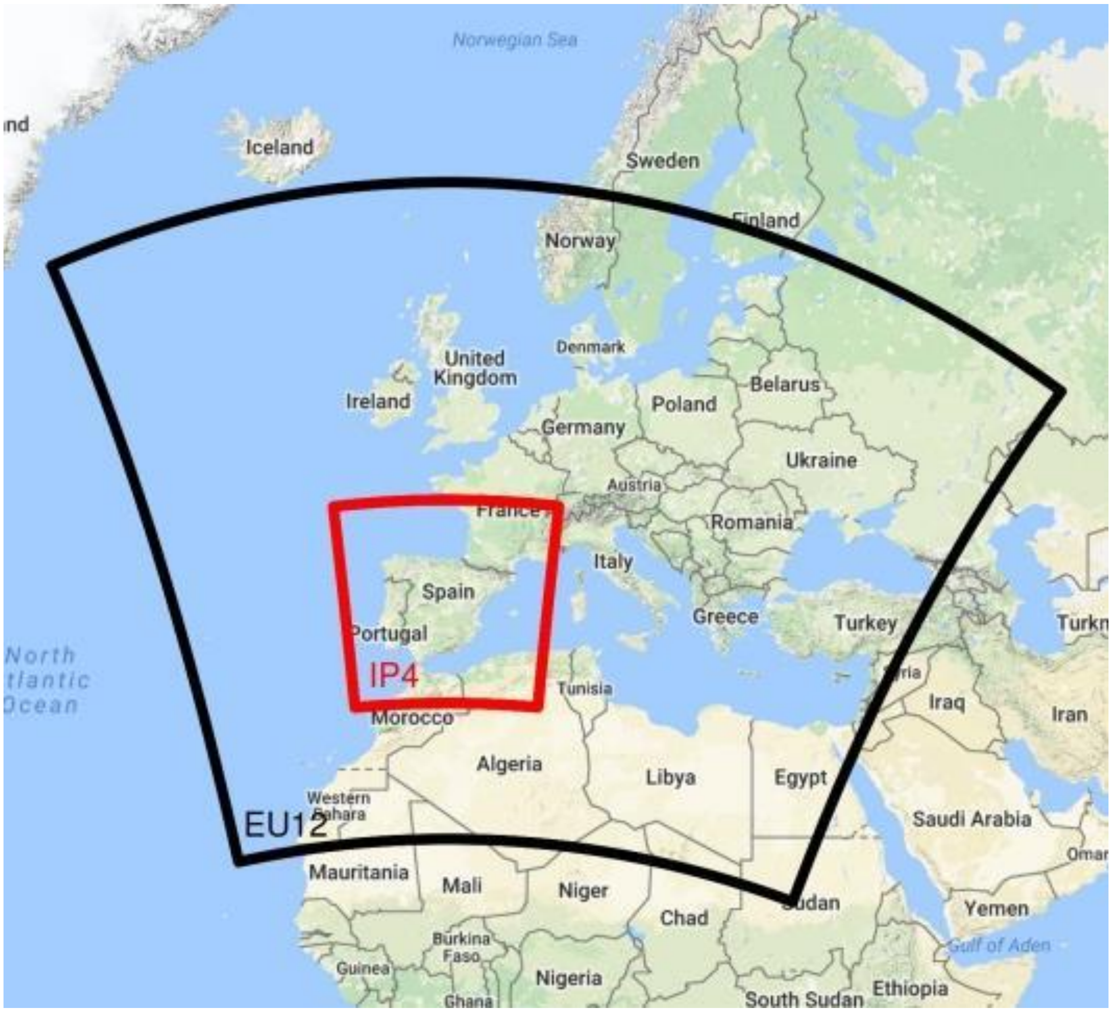

Figure S1. CMAQ domains in the CALIOPE system for the present study. EU12 corresponds to the mother domain at 12-km horizontal resolution (black). IP4 depicts the Iberian Peninsula domain at 4-km horizontal resolution used to run ISAM (red)

S2. Evaluation of biogenic emissions

Figure S2 shows the isoprene concentration at the Montseny station during the DAURE experimental campaign (Seco et al., 2011; http://cires.colorado.edu/jimenez-group/wiki/index.php/DAURE). Black dots indicate the measured isoprene concentration. Red lines indicate the modelled isoprene by MEGANv2.0.4 with global emission factor inputs files from MEGANv2.1. Blue lines show an experiment where emissions factors are based on local vegetation information. This evaluation indicates that modelled isoprene concentrations with updated emission factors are in reasonably good agreement with observations.

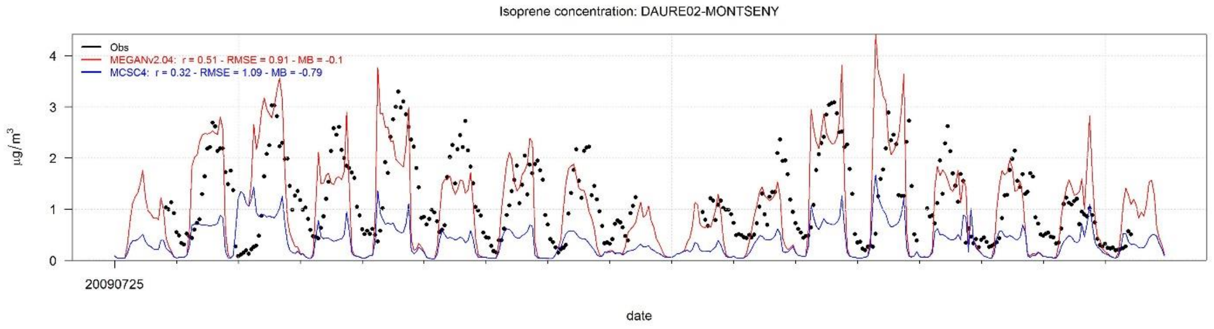

Figure S2. Time series for the isoprene concentration at the Montseny station during the DAURE experimental campaign

References:
Seco, R., Peñuelas, J., Filella, I., Llusià, J., Molowny-Horas, R., Schallhart, S., Metzger, A., Müller, M., and Hansel, A.: Contrasting winter and summer VOC mixing ratios at a forest site in the Western Mediterranean Basin: the effect of local biogenic emissions, Atmos. Chem. Phys., 11, 13161-13179, doi:10.5194/acp-11-13161-2011, 2011.

S3. Number of exceedances during the episode

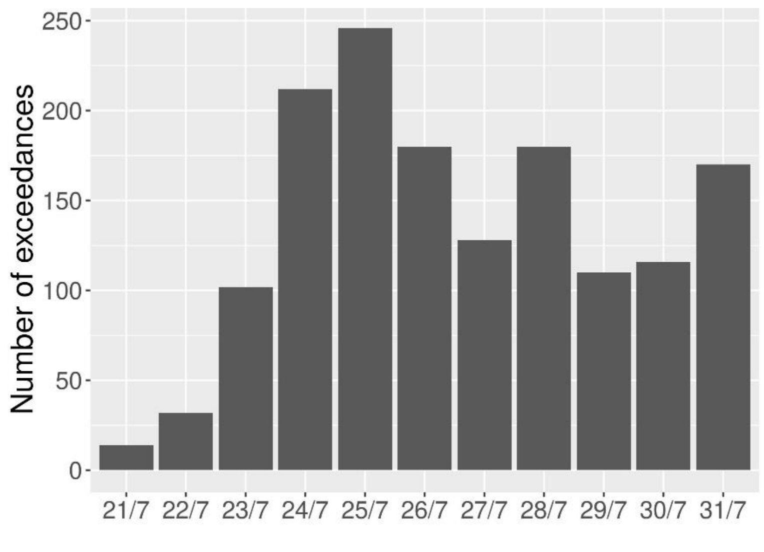

Figure S3. Number of stations exceeding the O_3 Target Value (120 µgm^{-3}) per episode day

S4. Model evaluation

We evaluate the updated version of CALIOPE using ISAM to quantify the system's ability to reproduce O_3 and NO_2 concentrations. We evaluate the simulated concentrations against air quality measurements from the Spanish monitoring stations that are part of the European Environment Information and

Observation Network (EIONET; https://www.eionet.europa.eu/). The EIONET network provides a relatively dense geographical coverage of the Spanish territory. During the July 21st-31st episode we used the measurements from 347 stations for O_3 and 357 stations for NO_2 with a temporal coverage above 85% on an hourly basis. Fig. S2 shows the distribution of the stations for O_3 and NO_2. Figure S4 depicts the MB, RMSE and *r* for average hourly and MDA8 O_3, and average hourly NO_2 concentrations.

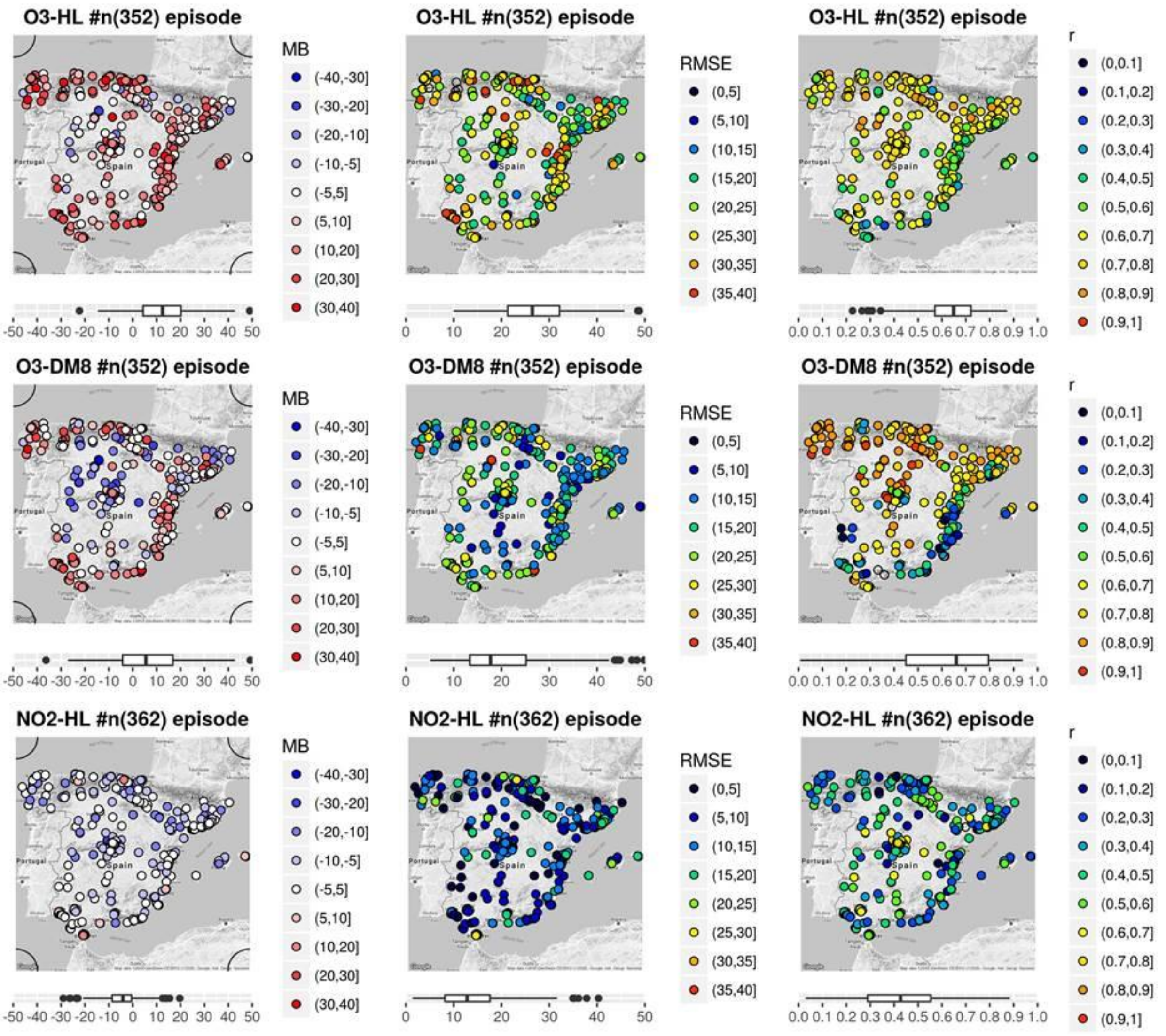

Figure S4. Mean bias (MB, in μg/m^3) (first column) , Root Mean Squared Error (RMSE, in μg/m^3) (second column) and correlation coefficient (r) (third column) for HL O_3, MDA8 O_3 and HL NO_2 at the Spanish EIONET stations during the selected O_3 episode

This section also evaluates the meteorological fields wind speed (WS), wind direction (WD) at 10 m and temperature at 2 m (T2M) using METeorological Aerodrome Report stations (METAR). For the selected episode, there were 50 METAR stations located at airports (see location in Fig. S5). Table S2 shows the summary of the statistical evaluation following the methodology explain in "Section 2.4 Evaluation method" for concentrations.

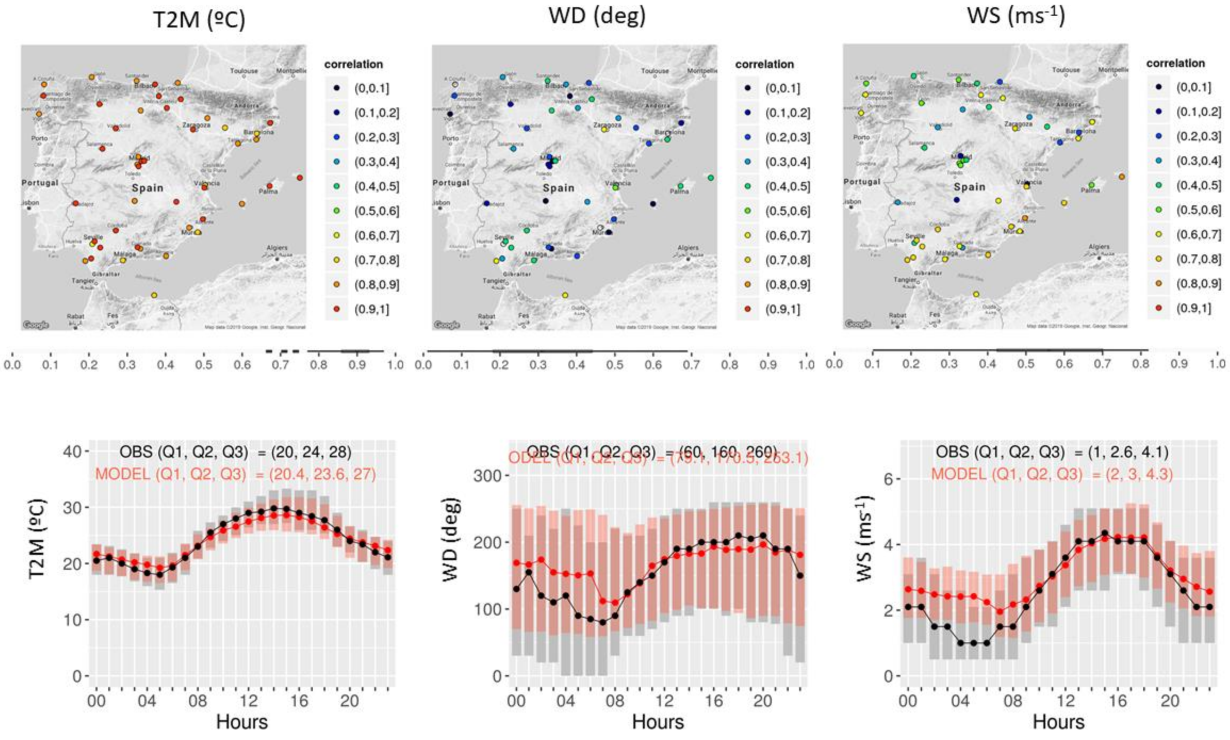

Figure S5. Hourly Temperature at 2 m (T2M, in ºC) (first column), Wind Direction (WD, in deg) (second column) and Wind Speed (WS) (third column) at the METAR (METeorological Aerodrome Report) stations over Spain during the selected O_3 episode. First row shows the correlation coefficient for each METAR station. Second row shows daily cycles for the meteorological variables for model and observations at the METAR stations. Q1, Q2 and Q3 indicate quartiles for the daily cycle. Bars show Q1 and Q3 at each hour

It is the modelled T2M that shows the best behaviour when compared with observations (r=0.91) (Table S2). The model slightly underestimates T2M (-0.2 ºC), especially for maximum and minimum temperatures (1.0ºC and 0.4 ºC for p25 and p75, respectively) (Fig. S5). The model reproduces the WS (r=0.42-0.70) with an overestimation of ~0.3 ms-1 on average. The overestimation is particularly marked during nighttime (Fig. S5), coincident with low-level wind speeds. These biases may contribute to the underestimation of surface concentrations of O3 precursors. The wind direction shows a lower correlation coefficient (0.1, 0.43). As for WS, daily cycle shows the better agreement with observation during the day and problems at nighttime.

Table S2. Statistics for T2M, WS and WD in the selected O_3 episode at the METAR stations. N indicates the number of pairs of data used in the discrete evaluation on an hourly basis and n the number of stations (see Fig. S2). Statistics are calculated by considering more than 75% of the hours in a day. The statistics correspond to following quantiles 50th (25th, 75th) by station.

	N/n	MO	MM	MB	NMB (%)	RMSE	r
T2M (ºC)	12924/50	25.0 (22.6,26.1)	24.4 (21.7,26.1)	-0.2 (-0.9, 0.2)	-0.9 (-4.5,0.9)	2.3 (1.8,2.7)	0.91 (0.86,0.93)
WD (deg)	12200/50	160.3 (131.8,186.9)	159.9 (140.4,209,5)	9.6 (-10.9,24.7)	6.2 (-7.1,16.3)	119.45 (101.6,141.5)	0.27 (0.1,0.43)
WS (ms^{-1})	13195/50	3.0 (2.5,3.3)	3.2 (2.9,3.5)	0.3 (-0.1,0.7)	12.3 (-3.9,26.1)	1.9 (1.6,2.1)	0.56 (0.42,0.70)

Overall, nighttime meteorology remains a challenge for meteorological models. The nighttime systematic overestimation of wind is a potential source of large error compensation for the modelling of NO2 and O3 nighttime concentrations.

References:

Vautard, R., Moran, M. D., Solazzo, E., Gilliam, R. C., Matthias, V., Bianconi, R., Chemel, C, Ferreira, J., Geyer, B., Hansen, A.B., Jericevic, A., Prank, M., Segers, A., Silver, J.D., Werhahn, J., Wolke, R., Rao, S.T., Galmarini, S.: Evaluation of the meteorological forcing used for the Air Quality Model Evaluation International Initiative (AQMEII) air quality simulations. Atmospheric Environment, 53, 15-37, 2012.

Bessagnet, B., Pirovano, G., Mircea, M., Cuvelier, C., Aulinger, A., Calori, G., Ciarelli, G., Manders, A., Stern, R., Tsyro, S., Gar- cía Vivanco, M., Thunis, P., Pay, M.-T., Colette, A., Couvidat, F., Meleux, F., Rouïl, L., Ung, A., Aksoyoglu, S., Baldasano, J. M., Bieser, J., Briganti, G., Cappelletti, A., D'Isidoro, M., Fi- nardi, S., Kranenburg, R., Silibello, C., Carnevale, C., Aas, W., Dupont, J.-C., Fagerli, H., Gonzalez, L., Menut, L., Prévôt, A. S. H., Roberts, P., and White, L.: Presentation of the EURODELTA III intercomparison exercise – evaluation of the chemistry transport models' performance on criteria pollutants and joint analysis with meteorology, Atmos. Chem. Phys., 16, 12667–12701, doi:10.5194/acp-16-12667-2016, 2016.

S5. Tagged NO_2 concentrations

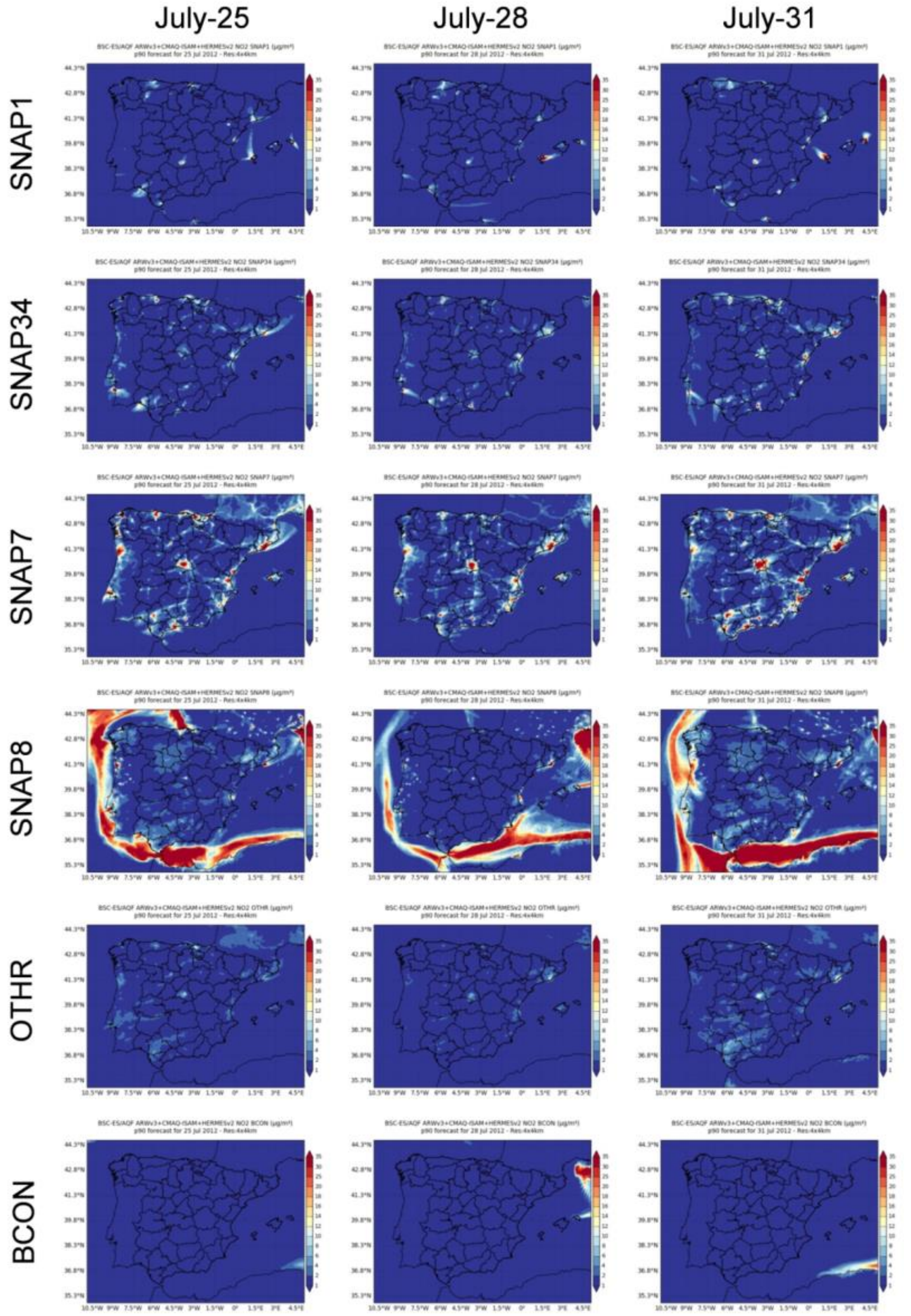

Figure S6. Tagged NO_2 concentrations (in μgm^{-3}) corresponding to the 90th percentile (90p) of the average hourly concentrations: SNAP1, SNAP34, SNAP7, SNAP8, OTHER, and BCON for July 25th (first column), 28th (second column) and 31st (third columns) in 2012

S6. Regionalization of source-sector contributions

Table S3. Absolute (μgm^{-3}) and normalized (%) contribution of tagged sources to surface O_3 concentration by receptor region: Center of the IP (CIP), Eastern IP (EIP), Ebro Valley (EV), Guadalquivir Valley (GV), the Mediterranean Sea (MED), North-Eastern IP (NEIP), Northern IP (NIP), North-Western IP (NWIP), Southern IP (SIP) and Western IP (WIP). Regions are sorted by decrease BCON O_3 concentration

Zone	*Concentration (μgm^{-3})*						*Percentage (%)*					
	BCON	*OTHR*	*SNAP1*	*SNAP34*	*SNAP7*	*SNAP8*	*BCON*	*OTHR*	*SNAP1*	*SNAP34*	*SNAP7*	*SNAP8*
MED	77.6	2.4	2.0	1.8	9.5	8.4	76	2	2	2	9	8
EV	67.7	3.7	3.2	3.2	10.8	11.6	68	4	3	3	11	12
EIP	67.1	3.7	2.8	2.9	12.2	11.9	67	4	3	3	12	12
NEIP	62.2	5.2	2.6	4.0	16.3	13.5	60	5	2	4	16	13
CIP	59.3	5.8	2.1	4.1	17.3	10.3	60	6	2	4	18	10
WIP	59.2	5.7	2.5	3.5	12.8	9.8	63	6	3	4	14	11
SIP	58.7	4.5	3.5	2.6	14.8	19.2	57	4	3	3	14	19
NIP	56.3	5.4	2.6	4.6	14.4	11.8	59	6	3	5	15	12
NWIP	55.7	5.8	4.4	4.5	13.6	10.3	59	6	5	5	14	11
GV	49.2	8.3	5.2	6.4	18.9	18.3	46	8	5	6	18	17

Note: We have excluded the ICON O_3 because its contribution is negligible after six days of spin-up.

S7. Ozone global maps

Surface	*Column*
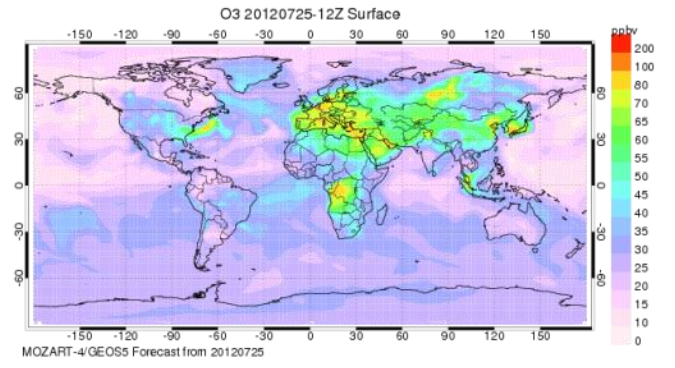	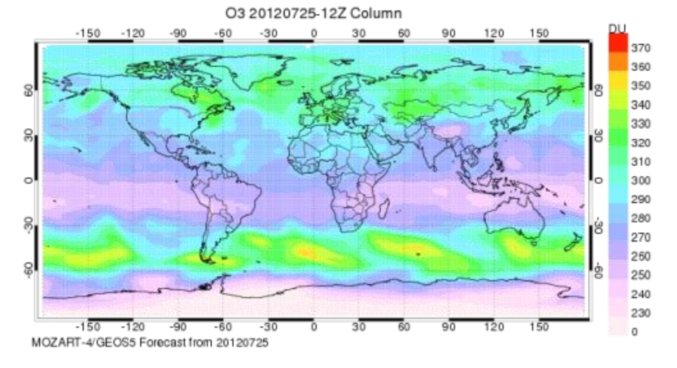
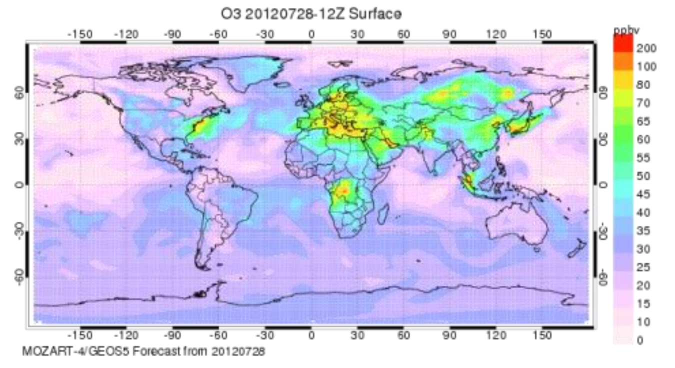	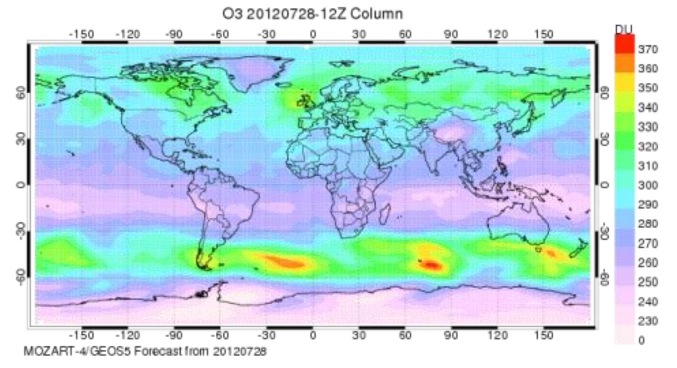
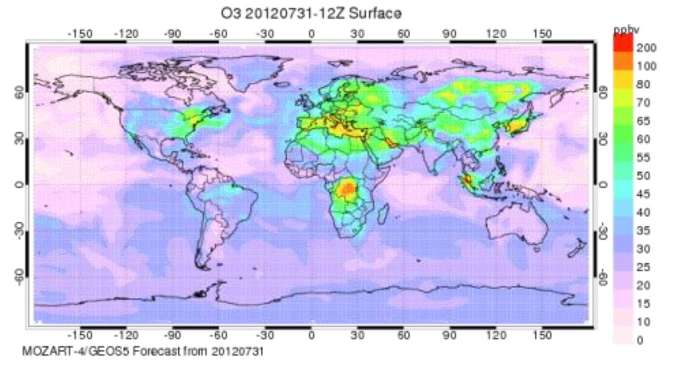	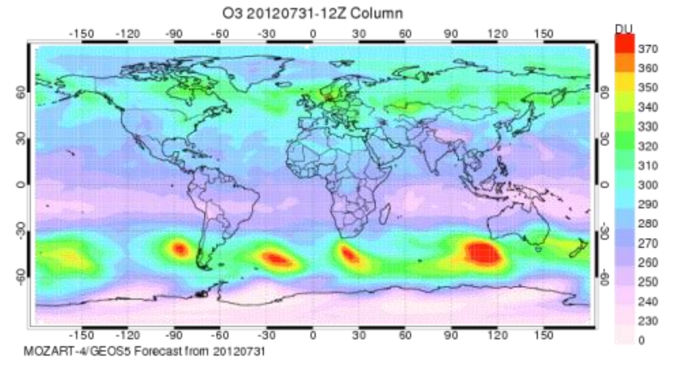

Figure S7. O_3 global maps for surface concentration (first column, in ppbv) and column (right column, in DU) for the July 25th (first row), 28th (second row) and 31st (third row) in 2012 for the MOZART-4 model used for CALIOPE boundary condition in the European domain (EU12). Source: http://www.acom.ucar.edu/acresp/forecast/
